# Glial cells as a promising therapeutic target of glaucoma: beyond the IOP

**DOI:** 10.3389/fopht.2023.1310226

**Published:** 2024-01-08

**Authors:** Youichi Shinozaki, Kazuhiko Namekata, Xiaoli Guo, Takayuki Harada

**Affiliations:** Visual Research Project, Tokyo Metropolitan Institute of Medical Science, Tokyo, Japan

**Keywords:** adeno-associated virus, astrocytes, cell transplantation, glaucoma, intraocular pressure, microglia, Müller cells

## Abstract

Glial cells, a type of non-neuronal cell found in the central nervous system (CNS), play a critical role in maintaining homeostasis and regulating CNS functions. Recent advancements in technology have paved the way for new therapeutic strategies in the fight against glaucoma. While intraocular pressure (IOP) is the most well-known modifiable risk factor, a significant number of glaucoma patients have normal IOP levels. Because glaucoma is a complex, multifactorial disease influenced by various factors that contribute to its onset and progression, it is imperative that we consider factors beyond IOP to effectively prevent or slow down the disease’s advancement. In the realm of CNS neurodegenerative diseases, glial cells have emerged as key players due to their pivotal roles in initiating and hastening disease progression. The inhibition of dysregulated glial function holds the potential to protect neurons and restore brain function. Consequently, glial cells represent an enticing therapeutic candidate for glaucoma, even though the majority of glaucoma research has historically concentrated solely on retinal ganglion cells (RGCs). In addition to the neuroprotection of RGCs, the proper regulation of glial cell function can also facilitate structural and functional recovery in the retina. In this review, we offer an overview of recent advancements in understanding the non-cell-autonomous mechanisms underlying the pathogenesis of glaucoma. Furthermore, state-of-the-art technologies have opened up possibilities for regenerating the optic nerve, which was previously believed to be incapable of regeneration. We will also delve into the potential roles of glial cells in the regeneration of the optic nerve and the restoration of visual function.

## 1 Introduction

Neurons in the mammalian central nervous system (CNS) have long been perceived as incapable of regeneration in the adult tissue (1). Similarly, retinal neurons have historically been considered non-regenerative, leading to the perception that blindness resulting from retinal neurodegenerative diseases and optic neuropathies is untreatable. Consequently, visual impairment in glaucoma, a leading global cause of blindness, has traditionally been thought of as irreversible. However, decades of extensive research have revealed that there is potential for restoring visual function even after the onset of ocular neurodegenerative diseases. Given that retinal ganglion cells (RGCs), responsible for transmitting visual information to the brain, are selectively damaged in glaucoma, therapeutic efforts have primarily focused on cell-autonomous mechanisms. These strategies have achieved significant success in preventing RGC death in glaucoma model animals and regenerating the optic nerve following optic nerve injuries. However, achieving a complete recovery of visual function remains a formidable challenge. To attain this goal, non-cell-autonomous mechanisms must also be considered, as numerous extrinsic factors play a role in regulating RGC degeneration and optic nerve regrowth. In this review, we delve into the pathogenic mechanisms of glaucoma and explore potential molecular targets for the restoration of visual function. We particularly focus on glial cells, a type of non-neuronal cell within the nervous system, as potential sources of these extrinsic factors.

## 2 Glaucoma

Glaucoma, progressive optic neuropathy, is the leading cause of blindness worldwide that affects more than 70 million people (2, 3). Despite the multifaceted nature of the disease, with numerous risk factors influencing its onset and progression (4), elevated intraocular pressure (IOP) is the most well-known and modifiable factor (5, 6). The scourge of blindness in glaucoma finds its genesis in the grievous impairment suffered by the optic nerve and RGCs. RGC degeneration is a hallmark of glaucoma (7), while the damage at the optic nerve head (ONH) — the part where RGC axons coalesce to form the optic nerve (8) — precedes the onset of visual field loss in glaucoma (9). Moreover, dendritic and synaptic degeneration in RGCs are also initial events that play a pivotal role in the progression of the disease (10, 11). Given the intrinsic limitations associated with the regenerative potential of both RGCs and optic nerves, extensive research endeavors have concentrated on the dual objectives of averting RGC demise and forestalling optic nerve degeneration, with the ultimate aim of reinstating visual function. A growing body of evidence has suggested that the regenerative capacity of the ocular tissue can be modifiable by various factors including the intracellular signaling molecules, extracellular factors, and environmental conditions. While interventions targeting cell-autonomous mechanisms have yielded substantial strides in optic nerve regeneration, the realization of comprehensive functional recovery remains a formidable challenge.

## 3 Heterogeneity in glial cells in the ocular tissue

**Figure 1:**
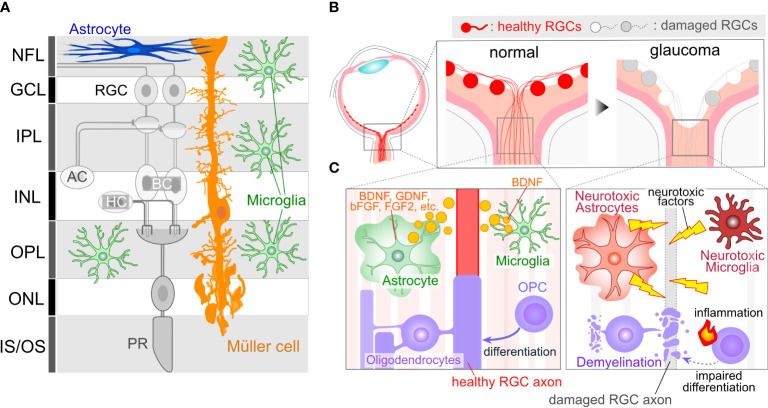
Glial Cells in Ocular Tissues. **(A)** Retinal Structure and Cellular Components: The retina comprises several neural layers. In the ganglion cell layer (GCL), one can find the cell bodies of RGCs. Additionally, some displaced amacrine cell (AC) somas are also localized within the GCL. RGC axons extend through the nerve fiber layer (NFL) and converge to form the optic nerve at the optic nerve head (ONH). Within the inner plexiform layer (IPL), RGC dendrites interact with axons from ACs or bipolar cells (BCs), forming synapses. Cell bodies for Acs and BCs reside in the inner nuclear layer (INL). Toward the outer part of the INL, horizontal cell (HC) bodies are present. In the outer plexiform layer (OPL), synapses formed by BCs, HCs, and photoreceptors (PRs) can be observed. PR cell bodies are located in the outer nuclear layer (ONL). PRs receive support from the retinal pigment epithelium, situated on the outer side of the PR inner/outer segments. Astrocytes are primarily found in the innermost retinal layer. Microglia are distributed across several retinal layers, including NFL/GCL, IPL, and OPL. Müller cells, which are retina-specific astrocyte-lineage cells, span vertically throughout the entire retinal thickness, with their cell bodies located in the INL, extending fine processes toward the synapses. **(B)** ONH Cupping in Glaucoma: ONH cupping represents well-characterized structural changes in the eyes of human glaucoma patients. These structural alterations may result in deformation and damage to RGC axons. Importantly, this change occurs in association with glial activations, suggesting that glial cells may contribute to the enlargement of cupping. **(C)** RGC axons in the ONH are unmyelinated and are directly enveloped by astrocytes and microglia. The ON is myelinated behind the optic nerve lamina region by OLs. OPCs also exist in the ON. Glial cells in normal conditions provide support for RGC axon integrity, including the production of neuroprotective factors (e.g. BDNF, GDNF, bFGF, and FGF2 from astrocytes and BDNF from microglia). However, in glaucoma, glial cells may undergo phenotypic changes, transitioning to neurotoxic states, which can lead to damage to RGC axons.

Glial cells, non-neuronal cell types in the nervous system, are not confined to the brain or spinal cord but also exist in the ocular tissue, such as the retina and optic nerve. In the retina, three types of glial cells exist: astrocytes, Müller cells, and microglia (**Figure 1A**). Astrocytes localize at the innermost surface of the retina and closely attach to blood vessels with their processes. Müller cells are the retina-specific astrocyte-lineage cells which are characterized by a vertical stalk spanning through the retina. Müller cells are limited to the retina, while astrocytes are highly enriched in the ONH and optic nerve (ON). Oligodendrocytes (OLs) and their precursors (oligodendrocyte precursor cells, OPCs) are present in the ON as illustrated in **Figure 1B**. RGC axon remain unmyelinated in most mammalian retina, with myelination commencing behind the myelination-transition zone located behind the globe. The myelination of the optic nerve facilitates the rapid transduction of visual information from the retina to the brain. Microglia, resident immune cells in the nervous tissues, including the retina, also contribute to the heterogeneity. Prior investigations have indicated that dysregulations in Müller cells and astrocytes can lead to RGC degeneration and visual dysfunction in the absence of elevated IOP (12, 13), underscoring their pivotal roles in the pathogenesis of normal tension glaucoma (NTG). Glial cells exhibit a remarkable degree of phenotypic plasticity, demonstrating either neurotoxic or neuroprotective attributes (14, 15). While it is widely acknowledged that reactive glial cells are frequently associated with neurotoxic functions, the appropriate regulation of glial cells has the potential to mitigate neuronal damage in a variety of neurodegenerative disease and CNS injury models (16–19). For instance, microglia-derived IL-1α, TNF, and C1q can induce the transformation of astrocytes into a neurotoxic phenotype, leading to damage to RGCs (14). Blockades, such as those preventing the formation of neurotoxic astrocytes, have been shown to protect RGCs in glaucoma model mice (20). On the other hand, microglia-derived TNFα, IL-1β, and IL-6 can induce astrocytes become reactive and neuroprotective (15). The neuroprotective effects of reactive astrocytes depend on STAT3 activity. Blocking the STAT3 signal in the astrocytes exacerbates RGC damage and visual impairment in the glaucoma model (21), emphasizing the crucial role of reactive astrocytes in protecting RGCs in glaucoma. Moreover, astrocytes and microglia tend to alter their phenotype in association with the disease state, exhibiting a relatively neuroprotective phenotype during the initial stages of neurodegenerative diseases in the brain (22, 23). In the case of the DBA/2J mouse, an inherited glaucoma model, reactive astrocytes have been found to confer protective effects upon RGCs during the early stages (23). Understanding these mechanisms and the precisely modulating glial cells represent an appealing avenue for neuroprotection. Alongside the phenotypic changes of glial cells, recent advancements in single-cell RNA sequencing (scRNA-seq) have unveiled heterogeneity among glial cells, revealing distinct subclusters that exhibit neuroprotective functions even under pathological conditions in the brain (24). While the most recent scRNA-sec data from the human and mouse retinas have successfully detected several subclasses of astrocytes, Müller cells, and microglia (25–29), these data are derived from human diseases or mouse models rather than specifically from glaucoma. Furthermore, in many cases of scRNA-seq data, ocular astrocytes and microglia are found as only a minor population and single cluster (13). Obtaining their subclusters in the scRNA-seq data requires cell isolation from at least several retinae (30). Given that scRNA-seq data from brain neurodegenerative disease model animals have detected disease-associated subclusters of glial cells (i.e. disease-associated microglia or astrocytes) that dynamically affect disease progression (31, 32), ocular glial cells in the human glaucoma patients or animal models would likely exhibit similar subclusters. Technical advancements in this field will enable us to uncover glaucoma-associated subclusters of glial cells and their role in glaucoma.

## 4 The Roles of glial cells in the enlargement of the ONH cupping

RGCs position their cell bodies within the ganglion cell layer (GCL), with their dendrites extending into the inner plexiform layer (IPL) (**Figure 1A**). GC axons converge to form the optic nerve at the ONH. The optic nerve exits the eye via the lamina cribrosa (LC), a mesh-like structure through which RGC axons pass. In human patients diagnosed with glaucoma, structural alterations in the LC lead to an enlargement of the ONH cupping (**Figure 1B**), a characteristic feature observable through ophthalmoscopy. Given that RGC axons passing through the LC are subject to deformation and damage due to ONH cupping, it becomes imperative to elucidate the cellular and molecular mechanisms underlying the pathogenesis of glaucoma. Rodents, frequently employed as experimental or genetic models for glaucoma, have traditionally been believed not to possess an LC structure that is rich in collagen, as is the case in humans (33). Instead, the equivalent region in rodents, known as the glial lamina, is highly enriched in astrocytes expressing glial fibrillary acidic protein (GFAP) (33). Given that the human LC also consists of astrocytes, and considering the close proximity of these astrocytes to RGC axons, any changes in their function are likely to exert a significant influence on the optic nerve (**Figure 1C**). In addition, the LC contains microglia, which become reactive and accumulate in response to optic nerve injuries (34). In humans, the LC is enriched in collagen, a major component of the extracellular matrix (ECM) (9). The enlargement of the ONH cupping is induced by ECM remodeling, a process initiated by degradation and production of ECM. Matrix metalloproteinases (MMPs), highly expressed in astrocytes and microglia, plays a role in ECM degradation (35, 36). Single nucleotide polymorphisms (SNPs) in the *MMP9* gene are associated with a higher risk of primary open-angle glaucoma (POAG) and NTG (37). Additionally, the production of ECM is crucial for tissue remodeling. Mutations or SNPs in ECM genes such as thrombospondin1 (*THBS1*) or fibronectin (*FNDC3B*) are linked to the risk of glaucoma (38, 39), and both are produced by astrocytes (40–42). Furthermore, since ONH cupping can be observed in patients irrespective of their IOP levels, including POAG and NTG, it is plausible that tissue changes and the pathogenesis of glaucoma are more closely linked to glial dysfunction than to elevated IOP.

## 5 The role of glial cells in promoting axonal regeneration within the ONH

**Figure 2:**
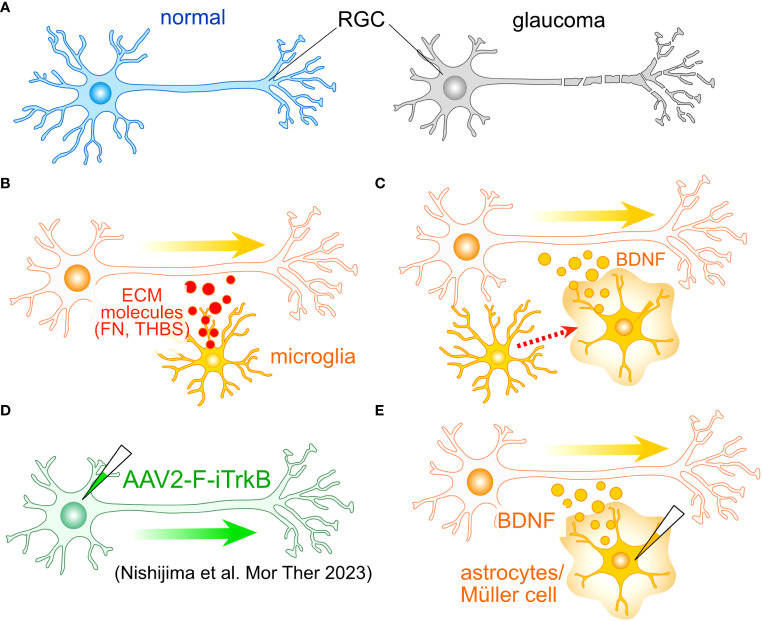
Potential roles of glial cells in the regeneration of RGC axon. **(A)** One of the most critical aspects of glaucoma is the damage to RGC axons. To identify potential molecular targets for axonal regeneration, researchers often employ the optic nerve crush (ONC) model. **(B)** Tissue regenerative microglia. Microglia involved in tissue regeneration express extracellular matrix (ECM) molecules like fibronectin (FN) and thrombospondin (THBS), potentially facilitating the regeneration of RGC axons. **(C)** Microglia-induced BDNF Expression: Microglia can induce the expression of BDNF in Müller cells, which may also accelerate axon regeneration. **(D)** An example of cell-autonomous enhancement of RGC axon regeneration. The induction of the farnesylated intracellular domain of TrkB (F-iTrkB) leads to a remarkable enhancement of axonal regeneration following ONC. **(E)** AAV-mediated expression of BDNF in astrocytes and Müller cells may stimulate axon regeneration and provide protection to RGCs, respectively.

Axonal injury occurring at the ONH stands as one of the pivotal events in the initiation and progression of glaucomatous pathology (**Figure 2A**). The optic nerve crush (ONC) model has been established as a well-recognized experimental paradigm for the assessment of axonal regeneration. Earlier investigations have unequivocally illustrated that axonal regeneration can be augmented through the grafting of peripheral nerves (43–45). This underscores the critical role played by extracellular factors and/or the microenvironment in regulating the regenerative capacity of RGC axons. In the context of spinal cord injury (SCI), the resurgence of axonal growth is contingent upon the presence of reactive astrocytes, while scar-forming astrocytes express molecules conducive to axonal growth, such as laminin (46). The effects are further potentiated by neurotrophic factors, which elicit a robust resurgence of axonal growth through the astrocytic scar and across lesion cores, exceeding control conditions by more than a hundredfold (47).

Microglia also harbor the potential to support axonal growth. The transplantation of immature microglia has been shown to significantly enhance the recuperative process and foster axon regeneration following SCI (48). These microglial cells manifest the expression of various ECM proteins, notably including fibronectin and thrombospondin (**Figure 2B**). Furthermore, they exhibit the presence of endopeptidase inhibitors, which serve as crucial regulators in the resolution of inflammation. The ECM-mediated facilitation of axonal regrowth is also instigated by astrocytes (46). Moreover, microglia have been observed to elicit the expression of brain-derived neurotrophic factor (BDNF) in Müller cells (49) (**Figure 2C**). Concurrent administration of neurotrophic factors alongside glia-mediated support has been demonstrated to engender a robust resurgence of axon growth following SCI (47). Consequently, the amalgamation of intrinsic mechanisms with glia-mediated support holds the promise of inducing a synergistic and remarkable rejuvenation of RGC axons.

## 6 The role of Müller cells in the protection of RGCs

Notably, neurotrophic factor signaling in reactive astrocytes has been documented to exert a protective influence on RGCs during the early stages of glaucoma (23). Among the neurotrophic factors, BDNF and its receptor TrkB are postulated to be pivotal in upholding the integrity of RGCs in glaucoma (50, 51). Müller cells emerge as the primary source of neurotrophic factors, their induction is triggered by various stimuli and insults (52–57). The sustained expression of BDNF in Müller cells has been demonstrated to confer protection upon RGCs following optic nerve injury (58, 59). Recent research has spotlighted the adeno-associated virus (AAV)-mediated enhancement of TrkB signaling in RGCs, leading to both cryoprotection against glaucoma and a vigorous resurgence of RGC axons (60) (**Figure 2D**). Collectively, these findings posit that Müller cell-derived neurotrophic factors, with particular emphasis on BDNF, hold paramount importance in protecting RGCs against glaucoma. Additionally, both astrocytes and Müller cells emerge as promising candidates for promoting the regeneration of RGC axons (**Figure 2E**).

## 7 Synapse disassembly in the context of glaucoma

**Figure 3:**
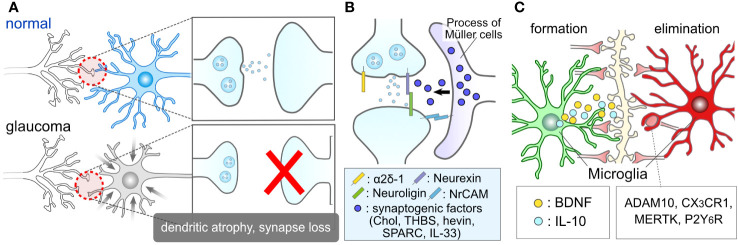
Glial Roles in Synapse Elimination and Formation. **(A)** Synaptic damage in glaucoma. Glaucoma results in the damage of RGC dendrites and retinal synapses. **(B)** Müller cell association with retinal synapses. The processes of Müller cells closely interact with retinal synapses. They might release synaptogenic factors, including cholesterol (Chol), thrombospondin (THBS), hevin, SPARC, interleukin-33 (IL-33), and regulate RGC synapses. **(C)** Microglial involvement in synapse formation and elimination. Microglia are recognized for their role in inducing synapse formation through contact and the release of factors such as BDNF and IL-10. Moreover, microglia are well-documented contributors to synapse elimination through various molecules, including ADAM10, CX3CR1, MERTK, and P2Y_6_R.

Glaucoma has traditionally been regarded as an optic neuropathy that results in optic nerve damage and RGC degeneration. RGC dendrites receive inputs from bipolar and amacrine cells, establishing synaptic connections in the IPL (**Figure 1A**). Visual information from photoreceptors is relayed to RGCs, then transmitted via the optic nerve to visual centers in the brain. Dendritic atrophy and synapse loss in RGCs can lead to visual deficits. It is well-established that RGCs undergo age-related dendritic atrophy preceding the degeneration of their cell bodies (61) (**Figure 3A**). Dendritic atrophy and synapse loss in RGCs represent shared structural characteristics observed in animal models of glaucoma and post-mortem human retinas (10, 11, 61–67). An accumulating body of evidence suggests that dendritic atrophy in RGCs and synapse loss within the IPL constitute early indicators of glaucomatous pathology (67–71), alongside optic nerve and RGC soma degeneration. Although achieving selective control over dendritic/synaptic atrophy poses a challenge, several studies have demonstrated that inhibiting atrophy is associated with the protection of RGC soma (11, 64, 72). Prevention of dendritic atrophy has been realized through various approaches, including blockade of the complement pathway (11), intravitreal injection of chondroitinase ABC (66) or BDNF (72). In glaucoma, synapses within the IPL are marked by complement C1q (70), and blocking the complement pathway has been shown to confer protection to RGCs (11, 73). Resident microglia eliminate the synapses with C1q and its downstream C3 (70). These extracellular signals and molecules appear to be promising targets for glaucoma treatment. Among them, ANX007, an anti-C1q monoclonal antibody, is currently undergoing clinical trials for the treatment of glaucoma (74).

## 8 The involvement of glial cells in synaptic maintenance

Glial cells play crucial roles in the regulation of synapses, both under normal physiological conditions and in pathological contexts. For instance, glial cells serve as important regulators during the critical period, which is a developmental stage characterized by heightened synaptic plasticity within the nervous system (75, 76). Astrocytes contribute to the formation of synapses through contact-mediated signaling (77, 78) and the production of synaptogenic factors, such as cholesterol (79), thrombospondin (80), hevin, SPARC (81), IL-33 (82) and neuronal adhesion molecule (83) (**Figure 3B**). While these factors were initially identified in brain astrocytes, they should also be expressed by ocular astrocytes and Müller cells, as evidenced by recent single-cell RNA sequencing data in the human retina, which shows high expression of these genes in astrocytes and Müller cells (84). Microglia also play a role in synaptogenesis through direct contact with synapses (85–87) and the secretion of molecules like interleukin 10 (IL-10) and BDNF (88–90) (**Figure 3C**). Müller cells serve as a source of BDNF in the retina and express or produce BDNF under various conditions and stimuli (49, 54, 57, 91, 92). The BDNF signal plays a regulatory role in the formation of dendrites in RGCs (93). Given that the fine processes of Müller cells intimately associate with RGC soma (94, 95), dendrites (96), and axons (97), BDNF derived from Müller cells is likely to have a significant impact on the regulation of RGC dendrites.

Glial cells also play a pivotal role in synapse elimination. Microglia, recognized as professional phagocytes, contribute to synapse elimination through various molecular mechanisms, including complement, ADAM10, CX3CR1, MERTK, and P2Y_6_ receptors (70, 98–103) (**Figure 3C**). Microglia-mediated synapse elimination serves as a crucial regulator in both the formation and maintenance of physiological neural circuits, as well as the disruption of pathological neural circuits. Dysfunctions in purinergic signaling, such as P2Y_6_ receptors, have been implicated in the pathogenesis of glaucoma (104–106). Additionally, astrocytes, considered non-professional phagocytes, also participate in synapse pruning through various factors like MEGF10 and MERTK (107–110). In the absence of microglia, astrocytes adopt phagocytic capabilities via TAM receptors (111). Beyond the individual responses of these cells, astrocytes and microglia coordinate their phagocytic functions (112). Moreover, bidirectional communication between them dynamically governs their functions and exerts an influence on synaptic and neuronal conditions (14, 15, 113, 114). These findings underscore the close relationship between glial conditions and synaptic conditions, highlighting glial cells as promising therapeutic targets in the context of glaucoma.

## 9 Oligodendrocyte dysfunction or loss in the context of glaucoma

Glial cells surrounding the optic nerve, such as OLs and OPC, may also play an important role in the pathogenesis of glaucoma. Myelin, formed by OLs, accelerates signal transduction through axons and provides essential energetic support. Deletion of the gene encoding myelin basic protein (MBP), highly expressed in OLs, leads to axonal swelling and degeneration (115). OLs exhibit persistent turn over, continuously replenished by newly differentiated cells from OPCs. Blocking OL turnover results in reduced myelination and axonal damage (116), highlighting the indispensability of OLs for axon homeostasis and functions.

Traditionally, glaucoma is not categorized as a demyelinating disease, however, emerging evidence suggests dysfunction and potential loss of OLs in glaucoma. DBA/2J mouse model demonstrates OL loss (117). A recent human study has indicated increased radial diffusivity within the optic radiations, serving as a surrogate marker for myelin damage (118). The study also observed a delay in the conduction of multifocal visual evoked potential, indicative of slowed conduction associated with myelin loss. In both the optic nerve injury (118) and glaucoma (119) animal models, OL loss and demyelination precede RGC damage. Maintaining OPC differentiation and myelination involves cell-autonomous mechanisms, such as thyroid hormone (120). Hypothyroidism is suggested as a risk factor for glaucoma (121–123), supporting the idea that impaired OL function contributes to the pathogenesis of glaucoma.

Additionally, non-cell-autonomous mechanisms may play a role. In the DBA/2J mouse, microglia in the myelinated region express and up-regulate the expression of Galectin-3/Mac-2, a phagocytosis-related gene (117), suggesting the involvement of microglia in myelin phagocytosis and the demyelination process. OPCs also express key phagocytotic genes and engage in axon pruning during the developing stage of mouse cortex (124), though the pathological consequence of OPC phagocytotic function in glaucoma remains unclear. OPCs may contribute to neuroinflammation and demyelination via low-density lipoprotein-related receptor 1 (LRP1) (125).

Another potential mechanism involves astrocyte-mediated cholesterol support (126). In the experimental autoimmune encephalomyelitis (EAE), a mouse model of multiple sclerosis (MS), astrocytes show down-regulated cholesterol synthesis and increased immune responses. Furthermore, phagocytosis by astrocytes may contribute to the demyelination (127, 128). Beyond demyelination, altered energetic support by OLs may be a crucial factor in glaucoma. In human patients with glaucoma, OL mitochondria are small (129). In DBA/2J mouse, monocarboxylate transporter 1 (MCT1), a lactate transporter, is down-regulated in OLs (130), suggesting reduced energetic support by OLs in glaucoma.

Preserving or restoring myelin could be a promising target for glaucoma treatment. Since cholesterol synthesis is promoted during remyelination (131), expediting cholesterol synthesis in astrocytes and/or oligodendrocytes may prove beneficial for glaucoma. Activation of astrocytic ABCA1 stimulates cholesterol synthesis (126) and supports oligodendrocyte survival and myelination (132). Astrocyte-derived CXCL1 also promotes remyelination by stimulating CXCR2 in OLs (133). A ketogenic diet might present an appealing approach to enhance energy availability by reversing the decline in MCT1 (130). Considering that neuronal activity boosts myelination (134, 135), visual stimulation could also be an attractive method for restoring RGC axons and visual function (136).

## 10 Tools for regulating glial cells and their potential role in glaucoma treatment

As mentioned earlier, glial cells have the capacity to influence the synapses, axons, and soma of RGCs, whether in a degenerative or regenerative manner. Beyond their neuroprotective capabilities, they hold significant potential for stimulating the regeneration of ocular structures and functions. In this section, we discuss various tools for controlling glial cell functions and their potential application in future glaucoma treatments. A summary of the advantages and disadvantages of each technique is shown in **Table 1**.

**Table 1:** Techniques for Glial Cell regulation: advantages and Disadvantages.

Techniques	Advantages	Disadvantages
1. AAV	a. Promoter and capsid-mediated cell specificity b. Prolonged therapeutic effect c. Already employed in clinical treatments	a'. Potential off-target effect b'. Impact on the innate immune system
2. PLX	a. Mainly affects microglia in the nervous system b. Well-regulated temporally c. Renewal and resetting of endogenous microglia	a'. Potential impact on border-associated macrophages and a subset of peripheral macrophages b'. Lacks tissue selectivity c'. Efficacy might be altered if microglial CSF1R expression were modified
3. Glial transplantation	a. iPSC-derived cells are applicable b. Grafted cells exhibit relatively long-term survival c. No need for immune suppression (via transnasal transplantation)	a'. May be influenced by the microenvironment of the host tissue b'. Lack tissue selectivity (via transnasal transplantation) c'. Invasive (injection-based transplantation)
4. TES	a. Already applied in clinical treatment b. Non-invasive c. Stimulation is selectively applied to the cornea	a'. Parameters should be optimized for glaucoma b'. Inappropriate settings may be detrimental to patients

### 10.1 Adeno-associated virus targeting glia for gene therapy in glaucoma

The genetic approach stands as a potent method for addressing neurodegenerative diseases, including ocular conditions (137). In addition to gene therapy aimed at neurons, targeting non-neuronal cells could also prove effective in treating glaucoma. Previously approved gene therapies have operated through non-cell-autonomous mechanisms. An exemplar is Luxturna™ (voretigene neparvovec-rzyl), the inaugural gene therapy approved for treating patients afflicted with inherited retinal dystrophy, a rare genetic disorder affecting the retina (138, 139). In this disease, blindness arises from photoreceptor (PR) degeneration, yet Luxturna™ targets the retinal pigment epithelium (RPE). The restoration of RPE functions provides support and protection to PRs. Such non-cell-autonomous mechanisms could similarly be applied to glaucoma. For cell type-specific gene therapy using AAV, specific promoters tailored for each cell type are employed. The glial fibrillary acidic protein (GFAP) promoter is a key promoter for astrocytes, and the gfaABC_1_D promoter, exhibiting nearly 100% specificity with 2-fold greater activity (140), is now widely adopted for precise astrocyte-specific gene manipulation via AAV. For achieving Müller cell specificity, there have been developments in engineering AAV capsids. Capsid variants derived from AAV6, such as ShH10 and ShH10Y, demonstrate efficient gene expression in Müller cells upon intravitreal injection (141–143). Moreover, the retinaldehyde-binding protein 1 (RLBP1) promoter has been successful in inducing gene expression specifically in Müller cells (143). For targeting OLs, promoters for the genes encoding proteolipid protein (144), myelin basic protein (145), and the myelin-associated glycoprotein (146) are used. Presently, foundational research efforts striving to facilitate the regeneration of RGC axons and synapses are categorized into two strategies: the promotion of regenerative factors and the prevention of inhibitory factors hindering regeneration. One example of the former strategy is BDNF. As previously mentioned, glial cells serve as the primary source of neurotrophic factors, including BDNF, and they maintain close associations with RGC axons and dendrites. The AAV-mediated expression of BDNF in astrocytes and Müller cells could potentially result in efficient delivery to axons and synapses, respectively. On the other hand, an example of the latter strategy involves insulin-like growth factor (IGF). Insulin and IGF share receptors and downstream signaling pathways (147) both of which are linked to RGC protection and the regeneration of dendrites, synapses, and axons (148–151). Notably, IGF-binding protein (IGFBP), which binds to IGF and hampers its signaling, becomes upregulated in astrocytes during neurodegenerative conditions and neurodevelopmental diseases (152–154). Given that the inhibition of astrocytic IGFBP partially restores neuronal function in the brain (152), the suppression of IGFBP signaling in astrocytes and/or Müller cells may prove beneficial for safeguarding and rejuvenating RGCs by enhancing IGF signaling.

**Figure 4:**
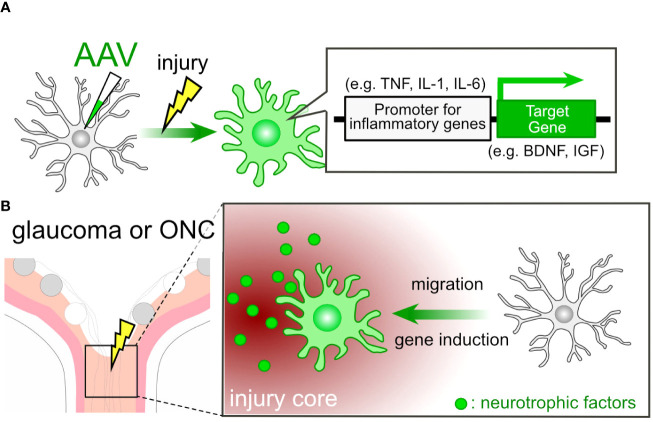
AAV-Mediated Cell Engineering and Target Molecule Delivery by Microglia. **(A)** AAV-mediated induction of neurotrophic factor genes in microglia. AAV-mediated engineering can induce the expression of neurotrophic factor genes in microglia under pathological conditions. By employing promoters associated with proinflammatory genes, microglia can produce neurotrophic factors like BDNF and IGF in response to pathological conditions. **(B)** Microglial Response in Glaucoma or ONC: In cases of glaucoma or ONC, the injury core triggers the microglial proinflammatory program, resulting in the induction of neurotrophic factor genes in microglia treated with AAV.

The use of targeted gene therapy in microglia presents itself as an appealing candidate for the treatment of glaucoma. Previously, inducing gene expression in microglia using AAV posed a challenge, but cutting-edge techniques now enable us to achieve such gene induction (155–157). Lin et al. initiated the evolution of the AAV capsid protein (AAV-cMG) in conjunction with the Cre-LoxP system, resulting in selective gene induction in microglia *in vivo* (28). Okada et al. utilized a 1.7-kb putative promoter region of the *Iba1* gene for inducing gene expression in microglia/macrophage cells (156). Young et al. achieved enhanced selectivity for microglia/tissue-resident macrophages by inserting a random 21-mer into the AAV9 capsid (157). It is well-established that microglia exhibit high motility and accumulate at injury sites following ONC (34). These inherent characteristics of microglia allow us to utilize them as vectors for delivering molecules to the site of injury. Furthermore, we can employ proinflammatory gene promoters to activate the expression of the target gene (**Figure 4A**). For instance, promoters associated with interleukins and tumor necrosis factor α (TNFα) can be employed within this system, given that these molecules are produced by microglia at the lesion core (15). In cases of glaucoma and post-ONC, the ONH sustains damage, leading to the accumulation of microglia at the injury core. This, in turn, triggers the proinflammatory program in AAV-treated microglia, subsequently inducing the production of target genes (**Figure 4B**). Promoting remyelination emerges as an appealing strategy for vision recovery in glaucoma. The induction of connexin (Cx) genes, such as Cx32 and Cx47, has been associated with a protective effect against leukodystrophy (158, 159), indicating a potential impact on optic nerve remyelination. Moreover, the deletion of the *Chrm1*gene, which encodes muscarinic receptor 1, a negative regulator of OPC differentiation, leads to increased myelination and axon density (116). The limitation of this technique for the clinical application might lie in the efficiency of AAV delivery to the target tissue and cells. For example, achieving efficient AAV delivery to the optic nerve remains a challenging issue.

### 10.2 Pharmacological tools for controlling microglia: PLX compounds

**Figure 5:**
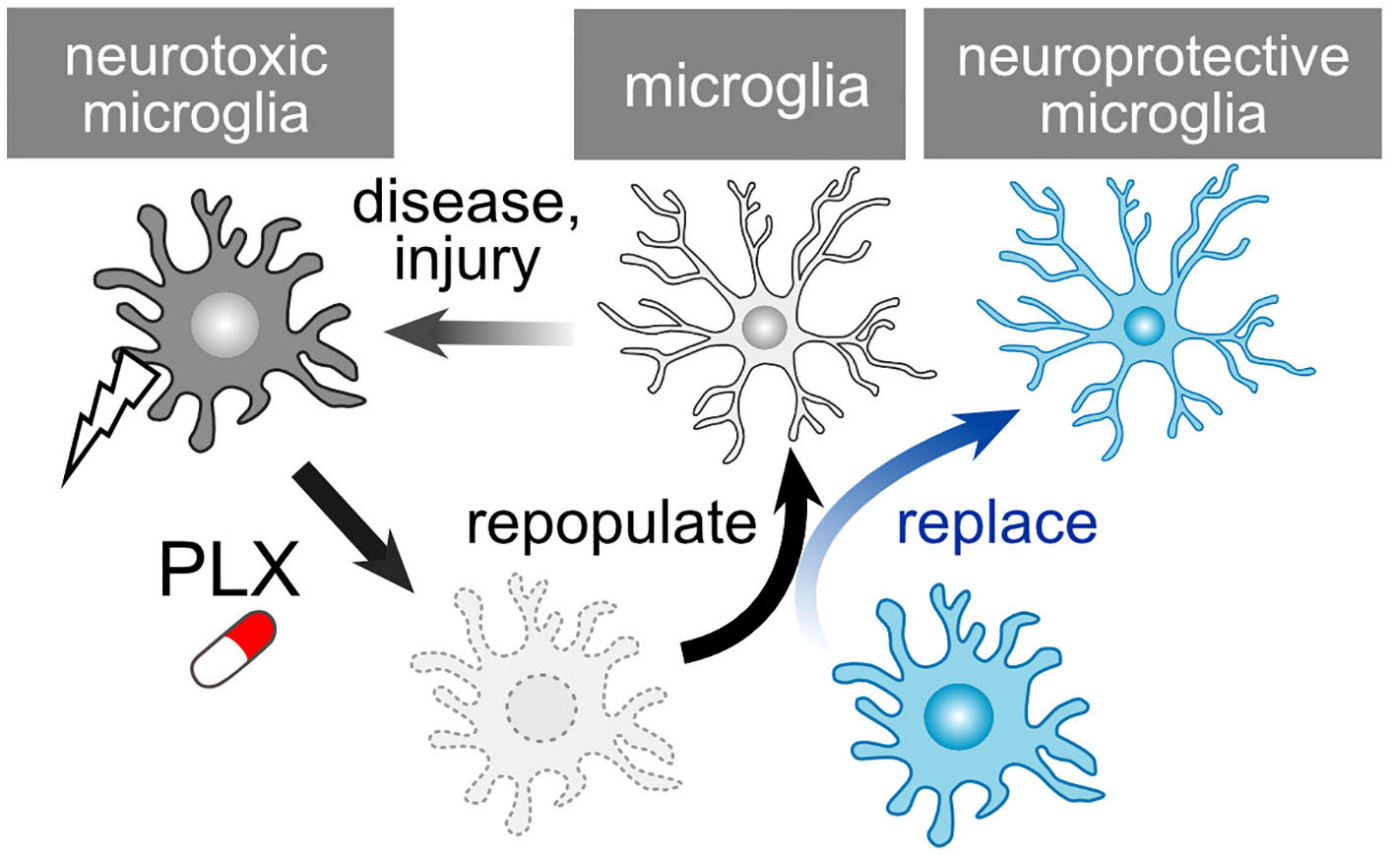
PLX-Mediated Repopulation and Replacement of Microglia. Microglia undergo a shift towards neurotoxic phenotypes following injury or in pathological conditions. PLX treatment depletes these microglia, and they subsequently repopulate once treatment is ceased. The newly generated microglia reset their neurotoxic characteristics. However, considering that repopulated microglia could revert to a neurotoxic phenotype, the replacement of microglia presents an additional strategy to maintain their health.

PLX compounds, originally developed by Plexxikon Inc., serve as potent antagonists for the colony-stimulating factor 1 receptor (CSF1R). Oral administration of PLX3397 for either 7 or 21 days results in a reduction in brain microglia numbers by 80-90% and over 95%, respectively (160). Several analogs of PLX compounds, including PLX3397, PLX5562, and PLX647, have been developed. Oral PLX treatment also leads to a significant decrease in the number of retinal microglia (161, 162). This effect is reversible, with microglia repopulating after the discontinuation of PLX compounds (**Figure 5**). In the case of retinal microglia, the rate of recovery varies among retinal layers, namely the NFL/GCL, IPL, and OPL (163). Upon removal of PLX, microglia spontaneously repopulate through proliferation in both the brain and retina. The removal and repopulation of microglia induce an anti-inflammatory response and promote brain recovery following injury (164–167). Numerous studies have demonstrated that microglia alter their phenotypes to become neurotoxic, and the removal and repopulation of microglia elicit a neuroprotective effect in models of neurodegenerative diseases, such as Alzheimer’s disease and Parkinson’s disease (168–171). Additionally, aside from brain diseases, microglia also play a role in neurodegenerative ocular injuries and diseases (172–174). Given that PLX treatment exhibits a protective effect on RGCs against *N*-methyl-D-aspartate (NMDA)-mediated toxicity (162), this compound may also have potential applications in the treatment of glaucoma. Although studies have shown that axonal regeneration after ONC is unaffected by the absence of microglia (175), this condition conceals both the neurodegenerative and supportive capabilities of microglia. PLX-mediated repopulation generates ‘new’ microglia with their phenotypes and functions reset, even in pathological conditions. For example, repopulation of aged microglia converts their cellular characteristics to a more youthful state, rescuing age-associated deficits in synapses and brain functions (176). Immature microglia possess the potential for anti-inflammatory responses and tissue regeneration (48). Such a ‘microglial reset’ could also prove valuable in restoring synapses and visual function in the context of glaucoma. Of note, PLX treatment can also be detrimental in certain situations. Microglia depletion from glaucoma model mice using PLX compounds exacerbates RGC damage (177, 178). Since microglia dynamically change their phenotypes, and there might be a neuroprotective glaucoma-associated microglial subcluster, techniques for more precise control of microglia are required, which could also pose a clinical limitation.

### 10.3 Cell transplantation

A recent study has demonstrated the feasibility of transplanting exogenous microglia into the CNS following microglial depletion induced by PLX compounds (179, 180). By combining the depletion of neurotoxic microglia with the transplantation of healthy, normal microglia, a replacement strategy can be employed (**Figure 5**). The depletion process appears to be crucial, as microglia extend their processes and establish their own territorial domains with an approximate diameter of 50 μm in both mouse and human brains (181). The absence of endogenous microglia permits the exogenously transplanted microglia to infiltrate and integrate into the nervous tissue. When combined with AAV-mediated functionalization of microglia (**Figure 4B**), this approach enables the precise delivery of specific molecules to designated sites, enhancing the efficiency of recovery while minimizing potential side effects. Transplantation can also be accomplished by introducing human iPS cell-derived microglia (iPSMG) into the mouse retina (182). Beyond microglia, intravitreal astrocyte transplantation may prove beneficial in safeguarding RGCs against damage induced by kainic acid (183). Additionally, transplantation of OPCs contributes to the neuroprotection and regeneration of the optic nerve. OPC transplantation has been shown to protect RGC in glaucoma model animal (184). Furthermore, the transplantation of OPC-rich neurospheres induces the myelination of the optic nerve (185–187). One limitation of clinical application may involve the duration for which the grafted cells survive in the host tissue. In the case of the iPSMG, transnasal transplantation to the brain maintained the grafted microglia for at least 60 days in mice (179, 180). Injected iPSMGs in the mouse retina survived over 200 days (182). However, it is not clear how long they would survive in the human tissue. Another concern is whether the grafted cells maintain their healthy phenotypes, as their phenotypes can be influenced by the microenvironment of the host tissue.

### 10.4 Transcorneal electrical stimulation

**Figure 6:**
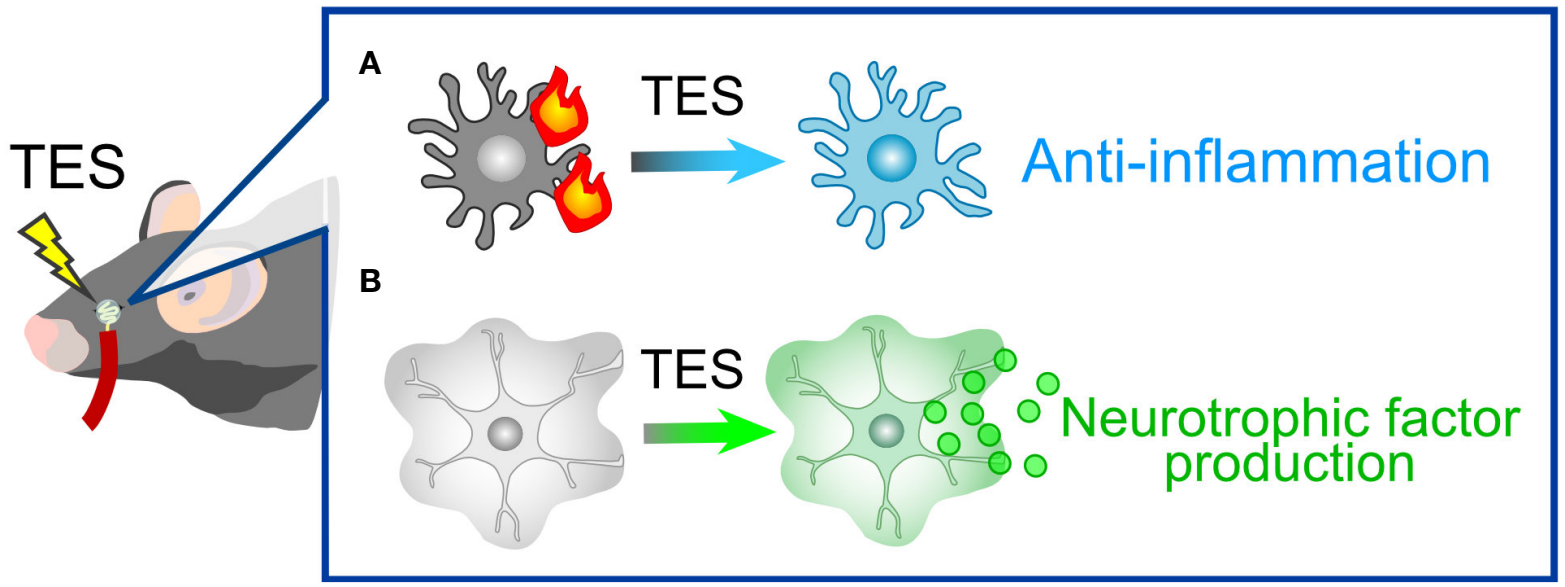
TES-Mediated Alterations in Glial Cells. **(A)** Microglial response. In pathological conditions, microglia tend to adopt pro-inflammatory phenotypes. TES effectively suppresses microglial inflammatory responses. **(B)** Müller Cell Expression: TES leads to an upregulation in the expression of neurotrophic factors, including FGF2, BDNF, and IGF, in Müller cells.

TES represents a non-invasive technique that administers electrical stimulation to the retina via the cornea. This approach has demonstrated therapeutic efficacy in both human patients and animal models afflicted with various injuries and diseases, including ischemic and traumatic optic neuropathies (188), axotomy (189, 190), retinal artery occlusion (191), ischemic damage (192), and photoreceptor degeneration (193, 194). TES has also exhibited a protective effect on RGCs in mouse models of glaucoma (195) and holds the potential to enhance visual function in human patients with glaucoma (196). While the precise mechanisms underlying TES are not fully elucidated, one of its neuroprotective mechanisms involves actions mediated by glial cells. For instance, TES suppresses pro-inflammatory responses by microglia (190, 197, 198) (**Figure 6A**). Simultaneously, TES induces the expression of various neurotrophic factors, including fibroblast growth factor 2 (FGF2), BDNF, and IGF, in Müller cells (54–56) (**Figure 6B**). The combination of anti-inflammatory effects and neurotrophic support is likely the primary mechanism behind RGC protection in glaucoma. As previously mentioned, these neurotrophic factors contribute not only to the protection of RGCs but also to the regeneration of dendrites, synapses, axons, and visual functions. Ongoing research aims to refine the parameters of TES for optimal neuroprotection and functional recovery (189, 199). TES is already employed in human patients with retinitis pigmentosa and has demonstrated safety and efficacy in improving visual function (200, 201). A current limitation of TES in the context of glaucoma treatment might be the absence of defined parameters. Optimized TES parameters will contribute to the development of a safe and effective treatment for glaucoma.

### 10.5 Extracellular vesicles

The EVs encompass membrane-derived vesicles with heterogenous groups, including exosomes and microvesicles (202). Initially described as a means to eliminate intracellular unneeded components to the extracellular space, subsequent studies have revealed their capacity for intercellular communication via transporting various molecules, including nucleic acids, lipids, and proteins. EVs are released from various cells and tissues including ocular cells and tissues. Due to their high stability and permeability to the blood-brain barrier (203), they represent attractive tools for drug delivery and hold significant potential as biomarkers for various diseases. In the ocular tissues, it has been reported that both retinal microglia and Müller cells release EVs (204–206). Considering the neuroprotective effects demonstrated by EVs from glial progenitors after traumatic brain injury (207), it is plausible that EVs from neuroprotective glial cells would similarly confer neuroprotection to RGCs. Moreover, embryonic stem cell-derived EVs, capable to delivering BDNF to other cells (208), could be employed as a therapeutic tool in glaucoma, for instance, by utilizing EVs derived from neurotrophin-overexpressing glia.

## 11 Concluding remarks

Glial cells are widely distributed throughout the nervous system, including ocular tissues. Pathological changes in glial cells play a pivotal role in driving RGC damage and resulting in visual deficits. Given that neuroprotective glia produce essential neurotrophic factors that impact both neuroprotection and neurodegeneration, glial cells represent an appealing therapeutic target for addressing glaucoma. With the aid of state-of-the-art techniques, we can precisely regulate glial functions, effectively suppressing neurotoxicity while enhancing neuroprotection and regeneration. Several glia-related molecules have already advanced to clinical trials, and we anticipate further advancements in drug discovery research aimed at targeting glial cells.

## Author contributions

YS: Funding acquisition, Validation, Visualization, Writing – original draft, Writing – review & editing. KN: Conceptualization, Validation, Writing – review & editing. XG: Funding acquisition, Validation, Writing – review & editing. TH: Project administration, Supervision, Validation, Writing – review & editing.

## References

[1] Illis LS. Central nervous system regeneration does not occur. Spinal Cord (2012) 50:259–63. doi: 10.1038/sc.2011.132 22105462

[2] Quigley HA. Number of people with glaucoma worldwide. Br J Ophthalmol (1996) 80:389–93. doi: 10.1136/bjo.80.5.389 PMC505485 8695555

[3] Weinreb RN Aung T Medeiros FA. The pathophysiology and treatment of glaucoma: a review. JAMA (2014) 311:1901–11. doi: 10.1001/jama.2014.3192 PMC4523637 24825645

[4] Weinreb RN Leung CK Crowston JG Medeiros FA Friedman DS Wiggs JL. Primary open-angle glaucoma. Nat Rev Dis Primers (2016) 2:16067. doi: 10.1038/nrdp.2016.67 27654570

[5] Heijl A Leske MC Bengtsson B Hyman L Bengtsson B Hussein M. Reduction of intraocular pressure and glaucoma progression: results from the Early Manifest Glaucoma Trial. Arch Ophthalmol (2002) 120:1268–79. doi: 10.1001/archopht.120.10.1268 12365904

[6] Kass MA Heuer DK Higginbotham EJ Johnson CA Keltner JL Miller JP. The Ocular Hypertension Treatment Study: a randomized trial determines that topical ocular hypotensive medication delays or prevents the onset of primary open-angle glaucoma. Arch Ophthalmol (2002) 120:701–13. doi: 10.1001/archopht.120.6.701 12049574

[7] Smith CA Vianna JR Chauhan BC. Assessing retinal ganglion cell damage. Eye (Lond) (2017) 31:209–17. doi: 10.1038/eye.2016.295 PMC5306472 28085141

[8] Quigley HA Addicks EM. Chronic experimental glaucoma in primates. II. Effect of extended intraocular pressure elevation on optic nerve head and axonal transport. Invest Ophthalmol Vis Sci (1980) 19:137–52. 6153173

[9] Sommer A Katz J Quigley HA Miller NR Robin AL Richter RC. Clinically detectable nerve fiber atrophy precedes the onset of glaucomatous field loss. Arch Ophthalmol (1991) 109:77–83. doi: 10.1001/archopht.1991.01080010079037 1987954

[10] Williams PA Howell GR Barbay JM Braine CE Sousa GL John SW. Retinal ganglion cell dendritic atrophy in DBA/2J glaucoma. PloS One (2013) 8:e72282. doi: 10.1371/journal.pone.0072282 23977271 PMC3747092

[11] Williams PA Tribble JR Pepper KW Cross SD Morgan BP Morgan JE. Inhibition of the classical pathway of the complement cascade prevents early dendritic and synaptic degeneration in glaucoma. Mol Neurodegener (2016) 11:26. doi: 10.1186/s13024-016-0091-6 27048300 PMC4822272

[12] Harada T Harada C Nakamura K Quah HM Okumura A Namekata K. The potential role of glutamate transporters in the pathogenesis of normal tension glaucoma. J Clin Invest (2007) 117:1763–70. doi: 10.1172/JCI30178 PMC1890997 17607354

[13] Shinozaki Y Leung A Namekata K Saitoh S Nguyen HB Takeda A. Astrocytic dysfunction induced by ABCA1 deficiency causes optic neuropathy. Sci Adv (2022) 8:eabq1081. doi: 10.1126/sciadv.abq1081 36332025 PMC9635836

[14] Liddelow SA Guttenplan KA Clarke LE Bennett FC Bohlen CJ Schirmer L. Neurotoxic reactive astrocytes are induced by activated microglia. Nature (2017) 541:481–7. doi: 10.1038/nature21029 PMC5404890 28099414

[15] Shinozaki Y Shibata K Yoshida K Shigetomi E Gachet C Ikenaka K. Transformation of astrocytes to a neuroprotective phenotype by microglia via P2Y(1) receptor downregulation. Cell Rep (2017) 19:1151–64. doi: 10.1016/j.celrep.2017.04.047 28494865

[16] Escartin C Galea E Lakatos A O’Callaghan JP Petzold GC Serrano-Pozo A. Reactive astrocyte nomenclature, definitions, and future directions. Nat Neurosci (2021) 24:312–25. doi: 10.1038/s41593-020-00783-4 PMC8007081 33589835

[17] Maragakis NJ Rothstein JD. Mechanisms of Disease: astrocytes in neurodegenerative disease. Nat Clin Pract Neurol (2006) 2:679–89. doi: 10.1038/ncpneuro0355 17117171

[18] Paolicelli RC Sierra A Stevens B Tremblay ME Aguzzi A Ajami B. Microglia states and nomenclature: A field at its crossroads. Neuron (2022) 110:3458–83. doi: 10.1016/j.neuron.2022.10.020 PMC9999291 36327895

[19] Perry VH Nicoll JA Holmes C. Microglia in neurodegenerative disease. Nat Rev Neurol (2010) 6:193–201. doi: 10.1038/nrneurol.2010.17 20234358

[20] Guttenplan KA Stafford BK El-Danaf RN Adler DI Munch AE Weigel MK. Neurotoxic reactive astrocytes drive neuronal death after retinal injury. Cell Rep (2020) 31:107776. doi: 10.1016/j.celrep.2020.107776 32579912 PMC8091906

[21] Sun D Moore S Jakobs TC. Optic nerve astrocyte reactivity protects function in experimental glaucoma and other nerve injuries. J Exp Med (2017) 214:1411–30. doi: 10.1084/jem.20160412 PMC5413323 28416649

[22] Fan Z Brooks DJ Okello A Edison P. An early and late peak in microglial activation in Alzheimer’s disease trajectory. Brain (2017) 140:792–803. doi: 10.1093/brain/aww349 28122877 PMC5837520

[23] Harder JM Braine CE Williams PA Zhu X MacNicoll KH Sousa GL. Early immune responses are independent of RGC dysfunction in glaucoma with complement component C3 being protective. Proc Natl Acad Sci U.S.A. (2017) 114:E3839–48. doi: 10.1073/pnas.1608769114 PMC5441748 28446616

[24] Choi I Wang M Yoo S Xu P Seegobin SP Li X. Autophagy enables microglia to engage amyloid plaques and prevents microglial senescence. Nat Cell Biol (2023) 25:963–74. doi: 10.1038/s41556-023-01158-0 PMC10950302 37231161

[25] Benhar I Ding J Yan W Whitney IE Jacobi A Sud M. Temporal single-cell atlas of non-neuronal retinal cells reveals dynamic, coordinated multicellular responses to central nervous system injury. Nat Immunol (2023) 24:700–13. doi: 10.1038/s41590-023-01437-w 36807640

[26] Krylov A Yu S Veen K Newton A Ye A Qin H. Heterogeneity in quiescent Muller glia in the uninjured zebrafish retina drive differential responses following photoreceptor ablation. Front Mol Neurosci (2023) 16:1087136. doi: 10.3389/fnmol.2023.1087136 37575968 PMC10413128

[27] Lamas M Martinez-Colin EJ. Muller cell molecular heterogeneity: facts and predictions. ASN Neuro (2022) 14:17590914221106903. doi: 10.1177/17590914221106903 35673270 PMC9184998

[28] Liu B He J Zhong L Huang L Gong B Hu J. Single-cell transcriptome reveals diversity of Muller cells with different metabolic-mitochondrial signatures in normal and degenerated macula. Front Neurosci (2022) 16:1079498. doi: 10.3389/fnins.2022.1079498 36620436 PMC9817153

[29] Monavarfeshani A Yan W Pappas C Odenigbo KA He Z Segre AV. Transcriptomic analysis of the ocular posterior segment completes a cell atlas of the human eye. Proc Natl Acad Sci U.S.A. (2023) 120:e2306153120. doi: 10.1073/pnas.2306153120 37566633 PMC10450437

[30] O’Koren EG Yu C Klingeborn M Wong AYW Prigge CL Mathew R. Microglial function is distinct in different anatomical locations during retinal homeostasis and degeneration. Immunity (2019) 50:723–737 e7. doi: 10.1016/j.immuni.2019.02.007 30850344 PMC6592635

[31] Deczkowska A Keren-Shaul H Weiner A Colonna M Schwartz M Amit I. Disease-associated microglia: A universal immune sensor of neurodegeneration. Cell (2018) 173:1073–81. doi: 10.1016/j.cell.2018.05.003 29775591

[32] Habib N McCabe C Medina S Varshavsky M Kitsberg D Dvir-Szternfeld R. Disease-associated astrocytes in Alzheimer’s disease and aging. Nat Neurosci (2020) 23:701–6. doi: 10.1038/s41593-020-0624-8 PMC9262034 32341542

[33] Howell GR Libby RT Jakobs TC Smith RS Phalan FC Barter JW. Axons of retinal ganglion cells are insulted in the optic nerve early in DBA/2J glaucoma. J Cell Biol (2007) 179:1523–37. doi: 10.1083/jcb.200706181 PMC2373494 18158332

[34] Qu J Jakobs TC. The time course of gene expression during reactive gliosis in the optic nerve. PloS One (2013) 8:e67094. doi: 10.1371/journal.pone.0067094 23826199 PMC3694957

[35] Cahoy JD Emery B Kaushal A Foo LC Zamanian JL Christopherson KS. A transcriptome database for astrocytes, neurons, and oligodendrocytes: a new resource for understanding brain development and function. J Neurosci (2008) 28:264–78. doi: 10.1523/JNEUROSCI.4178-07.2008 PMC6671143 18171944

[36] Yin KJ Cirrito JR Yan P Hu X Xiao Q Pan X. Matrix metalloproteinases expressed by astrocytes mediate extracellular amyloid-beta peptide catabolism. J Neurosci (2006) 26:10939–48. doi: 10.1523/JNEUROSCI.2085-06.2006 PMC6674654 17065436

[37] Suh W Won HH Kee C. The association of single-nucleotide polymorphisms in the MMP-9 gene with normal tension glaucoma and primary open-angle glaucoma. Curr Eye Res (2018) 43:534–8. doi: 10.1080/02713683.2017.1410177 29199866

[38] Fu H Siggs OM Knight LS Staffieri SE Ruddle JB Birsner AE. Thrombospondin 1 missense alleles induce extracellular matrix protein aggregation and TM dysfunction in congenital glaucoma. J Clin Invest (2022) 132:e156967. doi: 10.1172/JCI156967 36453543 PMC9711877

[39] Hysi PG Cheng CY Springelkamp H Macgregor S Bailey JNC Wojciechowski R. Genome-wide analysis of multi-ancestry cohorts identifies new loci influencing intraocular pressure and susceptibility to glaucoma. Nat Genet (2014) 46:1126–30. doi: 10.1038/ng.3087 PMC4177225 25173106

[40] Danjo Y Shigetomi E Hirayama YJ Kobayashi K Ishikawa T Fukazawa Y. Transient astrocytic mGluR5 expression drives synaptic plasticity and subsequent chronic pain in mice. J Exp Med (2022) 219:e20210989. doi: 10.1084/jem.20210989 35319723 PMC8952801

[41] Kim SK Hayashi H Ishikawa T Shibata K Shigetomi E Shinozaki Y. Cortical astrocytes rewire somatosensory cortical circuits for peripheral neuropathic pain. J Clin Invest (2016) 126:1983–97. doi: 10.1172/JCI82859 PMC4855913 27064281

[42] Tom VJ Doller CM Malouf AT Silver J. Astrocyte-associated fibronectin is critical for axonal regeneration in adult white matter. J Neurosci (2004) 24:9282–90. doi: 10.1523/JNEUROSCI.2120-04.2004 PMC6730112 15496664

[43] Benowitz LI Yin Y. Optic nerve regeneration. Arch Ophthalmol (2010) 128:1059–64. doi: 10.1001/archophthalmol.2010.152 PMC3072887 20697009

[44] Berry M Carlile J Hunter A. Peripheral nerve explants grafted into the vitreous body of the eye promote the regeneration of retinal ganglion cell axons severed in the optic nerve. J Neurocytol (1996) 25:147–70. doi: 10.1007/BF02284793 8699196

[45] Berry M Carlile J Hunter A Tsang W Rosenstiel P Sievers J. Optic nerve regeneration after intravitreal peripheral nerve implants: trajectories of axons regrowing through the optic chiasm into the optic tracts. J Neurocytol (1999) 28:721–41. doi: 10.1023/A:1007086004022 10859575

[46] Anderson MA Burda JE Ren Y Ao Y O’Shea TM Kawaguchi R. Astrocyte scar formation aids central nervous system axon regeneration. Nature (2016) 532:195–200. doi: 10.1038/nature17623 27027288 PMC5243141

[47] Anderson MA O’Shea TM Burda JE Ao Y Barlatey SL Bernstein AM. Required growth facilitators propel axon regeneration across complete spinal cord injury. Nature (2018) 561:396–400. doi: 10.1038/s41586-018-0467-6 30158698 PMC6151128

[48] Li Y He X Kawaguchi R Zhang Y Wang Q Monavarfeshani A. Microglia-organized scar-free spinal cord repair in neonatal mice. Nature (2020) 587:613–8. doi: 10.1038/s41586-020-2795-6 PMC7704837 33029008

[49] Harada T Harada C Kohsaka S Wada E Yoshida K Ohno S. Microglia-Muller glia cell interactions control neurotrophic factor production during light-induced retinal degeneration. J Neurosci (2002) 22:9228–36. doi: 10.1523/JNEUROSCI.22-21-09228.2002 PMC6758038 12417648

[50] Pease ME McKinnon SJ Quigley HA Kerrigan-Baumrind LA Zack DJ. Obstructed axonal transport of BDNF and its receptor TrkB in experimental glaucoma. Invest Ophthalmol Vis Sci (2000) 41:764–74. 10711692

[51] Quigley HA McKinnon SJ Zack DJ Pease ME Kerrigan-Baumrind LA Kerrigan DF. Retrograde axonal transport of BDNF in retinal ganglion cells is blocked by acute IOP elevation in rats. Invest Ophthalmol Vis Sci (2000) 41:3460–6. 11006239

[52] Honjo M Tanihara H Kido N Inatani M Okazaki K Honda Y. Expression of ciliary neurotrophic factor activated by retinal Muller cells in eyes with NMDA- and kainic acid-induced neuronal death. Invest Ophthalmol Vis Sci (2000) 41:552–60. 10670488

[53] Morimoto T Miyoshi T Matsuda S Tano Y Fujikado T Fukuda Y. Transcorneal electrical stimulation rescues axotomized retinal ganglion cells by activating endogenous retinal IGF-1 system. Invest Ophthalmol Vis Sci (2005) 46:2147–55. doi: 10.1167/iovs.04-1339 15914636

[54] Sato T Fujikado T Lee TS Tano Y. Direct effect of electrical stimulation on induction of brain-derived neurotrophic factor from cultured retinal Muller cells. Invest Ophthalmol Vis Sci (2008) 49:4641–6. doi: 10.1167/iovs.08-2049 18539944

[55] Sato T Fujikado T Morimoto T Matsushita K Harada T Tano Y. Effect of electrical stimulation on IGF-1 transcription by L-type calcium channels in cultured retinal Muller cells. Jpn J Ophthalmol (2008) 52:217–23. doi: 10.1007/s10384-008-0533-y 18661273

[56] Sato T Lee TS Takamatsu F Fujikado T. Induction of fibroblast growth factor-2 by electrical stimulation in cultured retinal Mueller cells. Neuroreport (2008) 19:1617–21. doi: 10.1097/WNR.0b013e3283140f25 18815585

[57] Seki M Tanaka T Sakai Y Fukuchi T Abe H Nawa H. Muller Cells as a source of brain-derived neurotrophic factor in the retina: noradrenaline upregulates brain-derived neurotrophic factor levels in cultured rat Muller cells. Neurochem Res (2005) 30:1163–70. doi: 10.1007/s11064-005-7936-7 16292510

[58] Di Polo A Aigner LJ Dunn RJ Bray GM Aguayo AJ. Prolonged delivery of brain-derived neurotrophic factor by adenovirus-infected Muller cells temporarily rescues injured retinal ganglion cells. Proc Natl Acad Sci U.S.A. (1998) 95:3978–83. doi: 10.1073/pnas.95.7.3978 PMC19948 9520478

[59] Mansour-Robaey S Clarke DB Wang YC Bray GM Aguayo AJ. Effects of ocular injury and administration of brain-derived neurotrophic factor on survival and regrowth of axotomized retinal ganglion cells. Proc Natl Acad Sci U.S.A. (1994) 91:1632–6. doi: 10.1073/pnas.91.5.1632 PMC43217 8127857

[60] Nishijima E Honda S Kitamura Y Namekata K Kimura A Guo X. Vision protection and robust axon regeneration in glaucoma models by membrane-associated Trk receptors. Mol Ther (2023) 31:810–24. doi: 10.1016/j.ymthe.2022.11.018 PMC10014229 36463402

[61] Samuel MA Zhang Y Meister M Sanes JR. Age-related alterations in neurons of the mouse retina. J Neurosci (2011) 31:16033–44. doi: 10.1523/JNEUROSCI.3580-11.2011 PMC3238393 22049445

[62] Berry RH Qu J John SW Howell GR Jakobs TC. Synapse loss and dendrite remodeling in a mouse model of glaucoma. PloS One (2015) 10:e0144341. doi: 10.1371/journal.pone.0144341 26637126 PMC4670161

[63] Jakobs TC Libby RT Ben Y John SW Masland RH. Retinal ganglion cell degeneration is topological but not cell type specific in DBA/2J mice. J Cell Biol (2005) 171:313–25. doi: 10.1083/jcb.200506099 PMC2171185 16247030

[64] Risner ML Pasini S McGrady NR Calkins DJ. Bax contributes to retinal ganglion cell dendritic degeneration during glaucoma. Mol Neurobiol (2022) 59:1366–80. doi: 10.1007/s12035-021-02675-5 PMC8882107 34984584

[65] Tribble JR Vasalauskaite A Redmond T Young RD Hassan S Fautsch MP. Midget retinal ganglion cell dendritic and mitochondrial degeneration is an early feature of human glaucoma. Brain Commun (2019) 1:fcz035. doi: 10.1093/braincomms/fcz035 31894207 PMC6928391

[66] Tribble JR Williams PA Caterson B Sengpiel F Morgan JE. Digestion of the glycosaminoglycan extracellular matrix by chondroitinase ABC supports retinal ganglion cell dendritic preservation in a rodent model of experimental glaucoma. Mol Brain (2018) 11:69. doi: 10.1186/s13041-018-0412-5 30463575 PMC6249825

[67] Weber AJ Kaufman PL Hubbard WC. Morphology of single ganglion cells in the glaucomatous primate retina. Invest Ophthalmol Vis Sci (1998) 39:2304–20. 9804139

[68] El-Danaf RN Huberman AD. Characteristic patterns of dendritic remodeling in early-stage glaucoma: evidence from genetically identified retinal ganglion cell types. J Neurosci (2015) 35:2329–43. doi: 10.1523/JNEUROSCI.1419-14.2015 PMC6605614 25673829

[69] Ou Y Jo RE Ullian EM Wong RO Della Santina L. Selective vulnerability of specific retinal ganglion cell types and synapses after transient ocular hypertension. J Neurosci (2016) 36:9240–52. doi: 10.1523/JNEUROSCI.0940-16.2016 PMC5005727 27581463

[70] Stevens B Allen NJ Vazquez LE Howell GR Christopherson KS Nouri N. The classical complement cascade mediates CNS synapse elimination. Cell (2007) 131:1164–78. doi: 10.1016/j.cell.2007.10.036 18083105

[71] Weber AJ Harman CD. Structure-function relations of parasol cells in the normal and glaucomatous primate retina. Invest Ophthalmol Vis Sci (2005) 46:3197–207. doi: 10.1167/iovs.04-0834 PMC1351226 16123419

[72] Weber AJ Harman CD. BDNF preserves the dendritic morphology of alpha and beta ganglion cells in the cat retina after optic nerve injury. Invest Ophthalmol Vis Sci (2008) 49:2456–63. doi: 10.1167/iovs.07-1325 18263808

[73] Bosco A Anderson SR Breen KT Romero CO Steele MR Chiodo VA. Complement C3-targeted gene therapy restricts onset and progression of neurodegeneration in chronic mouse glaucoma. Mol Ther (2018) 26:2379–96. doi: 10.1016/j.ymthe.2018.08.017 PMC6171099 30217731

[74] Sun Y Wirta D Murahashi W Mathur V Sankaranarayanan S Taylor LK. Safety and target engagement of complement C1q inhibitor ANX007 in neurodegenerative eye disease: results from phase I studies in glaucoma. Ophthalmol Sci (2023) 3:100290. doi: 10.1016/j.xops.2023.100290 37124168 PMC10130689

[75] Ackerman SD Perez-Catalan NA Freeman MR Doe CQ. Astrocytes close a motor circuit critical period. Nature (2021) 592:414–20. doi: 10.1038/s41586-021-03441-2 PMC9901311 33828296

[76] Ribot J Breton R Calvo CF Moulard J Ezan P Zapata J. Astrocytes close the mouse critical period for visual plasticity. Science (2021) 373:77–81. doi: 10.1126/science.abf5273 34210880

[77] Filosa A Paixao S Honsek SD Carmona MA Becker L Feddersen B. Neuron-glia communication via EphA4/ephrin-A3 modulates LTP through glial glutamate transport. Nat Neurosci (2009) 12:1285–92. doi: 10.1038/nn.2394 PMC3922060 19734893

[78] Murai KK Nguyen LN Irie F Yamaguchi Y Pasquale EB. Control of hippocampal dendritic spine morphology through ephrin-A3/EphA4 signaling. Nat Neurosci (2003) 6:153–60. doi: 10.1038/nn994 12496762

[79] Mauch DH Nagler K Schumacher S Goritz C Muller EC Otto A. CNS synaptogenesis promoted by glia-derived cholesterol. Science (2001) 294:1354–7. doi: 10.1126/science.294.5545.1354 11701931

[80] Christopherson KS Ullian EM Stokes CC Mullowney CE Hell JW Agah A. Thrombospondins are astrocyte-secreted proteins that promote CNS synaptogenesis. Cell (2005) 120:421–33. doi: 10.1016/j.cell.2004.12.020 15707899

[81] Kucukdereli H Allen NJ Lee AT Feng A Ozlu MI Conatser LM. Control of excitatory CNS synaptogenesis by astrocyte-secreted proteins Hevin and SPARC. Proc Natl Acad Sci U.S.A. (2011) 108:E440–9. doi: 10.1073/pnas.1104977108 PMC3156217 21788491

[82] Wang Y Fu WY Cheung K Hung KW Chen C Geng H. Astrocyte-secreted IL-33 mediates homeostatic synaptic plasticity in the adult hippocampus. Proc Natl Acad Sci U.S.A. (2021) 118:e2020810118. doi: 10.1073/pnas.2020810118 33443211 PMC7817131

[83] Takano T Wallace JT Baldwin KT Purkey AM Uezu A Courtland JL. Chemico-genetic discovery of astrocytic control of inhibition in vivo. Nature (2020) 588:296–302. doi: 10.1038/s41586-020-2926-0 33177716 PMC8011649

[84] Lukowski SW Patel J Andersen SB Sim SL Wong HY Tay J. Single-cell transcriptional profiling of aortic endothelium identifies a hierarchy from endovascular progenitors to differentiated cells. Cell Rep (2019) 27:2748–2758 e3. doi: 10.1016/j.celrep.2019.04.102 31141696

[85] Gallo NB Berisha A Van Aelst L. Microglia regulate chandelier cell axo-axonic synaptogenesis. Proc Natl Acad Sci U.S.A. (2022) 119:e2114476119. doi: 10.1073/pnas.2114476119 35263225 PMC8931231

[86] Miyamoto A Wake H Ishikawa AW Eto K Shibata K Murakoshi H. Microglia contact induces synapse formation in developing somatosensory cortex. Nat Commun (2016) 7:12540. doi: 10.1038/ncomms12540 27558646 PMC5007295

[87] Weinhard L di Bartolomei G Bolasco G MaChado P Schieber NL Neniskyte U. Microglia remodel synapses by presynaptic trogocytosis and spine head filopodia induction. Nat Commun (2018) 9:1228. doi: 10.1038/s41467-018-03566-5 29581545 PMC5964317

[88] Huang L Jin J Chen K You S Zhang H Sideris A. BDNF produced by cerebral microglia promotes cortical plasticity and pain hypersensitivity after peripheral nerve injury. PloS Biol (2021) 19:e3001337. doi: 10.1371/journal.pbio.3001337 34292944 PMC8346290

[89] Lim SH Park E You B Jung Y Park AR Park SG. Neuronal synapse formation induced by microglia and interleukin 10. PloS One (2013) 8:e81218. doi: 10.1371/journal.pone.0081218 24278397 PMC3838367

[90] Parkhurst CN Yang G Ninan I Savas JN Yates JR 3rd Lafaille JJ. Microglia promote learning-dependent synapse formation through brain-derived neurotrophic factor. Cell (2013) 155:1596–609. doi: 10.1016/j.cell.2013.11.030 PMC4033691 24360280

[91] Le YZ Xu B Chucair-Elliott AJ Zhang H Zhu M Mediates Retinal Muller Cell Viability VEGF. and neuroprotection through BDNF in diabetes. Biomolecules (2021) 11:712. doi: 10.3390/biom11050712 34068807 PMC8150851

[92] Suzumura A Kaneko H Funahashi Y Takayama K Nagaya M Ito S. n-3 fatty acid and its metabolite 18-HEPE ameliorate retinal neuronal cell dysfunction by enhancing muller BDNF in diabetic retinopathy. Diabetes (2020) 69:724–35. doi: 10.2337/db19-0550 32029482

[93] Lom B Cogen J Sanchez AL Vu T Cohen-Cory S. Local and target-derived brain-derived neurotrophic factor exert opposing effects on the dendritic arborization of retinal ganglion cells in vivo. J Neurosci (2002) 22:7639–49. doi: 10.1523/JNEUROSCI.22-17-07639.2002 PMC6757994 12196587

[94] Danjo Y Shinozaki Y Natsubori A Kubota Y Kashiwagi K Tanaka KF. The mlc1 promoter directs muller cell-specific gene expression in the retina. Transl Vis Sci Technol (2022) 11:25. doi: 10.1167/tvst.11.1.25 PMC8764212 35040915

[95] Wang J O’Sullivan ML Mukherjee D Punal VM Farsiu S Kay JN. Anatomy and spatial organization of Muller glia in mouse retina. J Comp Neurol (2017) 525:1759–77. doi: 10.1002/cne.24153 PMC5542564 27997986

[96] Tworig JM Coate CJ Feller MB. Excitatory neurotransmission activates compartmentalized calcium transients in Muller glia without affecting lateral process motility. Elife (2021) 10:e73202. doi: 10.7554/eLife.73202 34913435 PMC8806189

[97] Shinozaki Y Kashiwagi K Koizumi S. Astrocyte immune functions and glaucoma. Int J Mol Sci (2023) 24:2747. doi: 10.3390/ijms24032747 PMC9916878 36769067

[98] Dundee JM Puigdellivol M Butler R Cockram TOJ Brown GC. P2Y(6) receptor-dependent microglial phagocytosis of synapses mediates synaptic and memory loss in aging. Aging Cell (2023) 22:e13761. doi: 10.1111/acel.13761 36565471 PMC9924939

[99] Gunner G Cheadle L Johnson KM Ayata P Badimon A Mondo E. Sensory lesioning induces microglial synapse elimination via ADAM10 and fractalkine signaling. Nat Neurosci (2019) 22:1075–88. doi: 10.1038/s41593-019-0419-y PMC6596419 31209379

[100] Paolicelli RC Bolasco G Pagani F Maggi L Scianni M Panzanelli P. Synaptic pruning by microglia is necessary for normal brain development. Science (2011) 333:1456–8. doi: 10.1126/science.1202529 21778362

[101] Park J Choi Y Jung E Lee SH Sohn JW Chung WS. Microglial MERTK eliminates phosphatidylserine-displaying inhibitory post-synapses. EMBO J (2021) 40:e107121. doi: 10.15252/embj.2020107121 34013588 PMC8327958

[102] Schafer DP Lehrman EK Kautzman AG Koyama R Mardinly AR Yamasaki R. Microglia sculpt postnatal neural circuits in an activity and complement-dependent manner. Neuron (2012) 74:691–705. doi: 10.1016/j.neuron.2012.03.026 22632727 PMC3528177

[103] Wang C Yue H Hu Z Shen Y Ma J Li J. Microglia mediate forgetting via complement-dependent synaptic elimination. Science (2020) 367:688–94. doi: 10.1126/science.aaz2288 32029629

[104] Hamada K Shinozaki Y Namekata K Matsumoto M Ohno N Segawa T. Loss of P2Y(1) receptors triggers glaucoma-like pathology in mice. Br J Pharmacol (2021) 178:4552–71. doi: 10.1111/bph.15637 34309010

[105] Shinozaki Y Kashiwagi K Namekata K Takeda A Ohno N Robaye B. Purinergic dysregulation causes hypertensive glaucoma-like optic neuropathy. JCI Insight (2017) 2:e93456. doi: 10.1172/jci.insight.93456 28978804 PMC5841869

[106] Shinozaki Y Saito K Kashiwagi K Koizumi S. Ocular P2 receptors and glaucoma. Neuropharmacology (2023) 222:109302. doi: 10.1016/j.neuropharm.2022.109302 36341810

[107] Byun YG Kim NS Kim G Jeon YS Choi JB Park CW. Stress induces behavioral abnormalities by increasing expression of phagocytic receptor MERTK in astrocytes to promote synapse phagocytosis. Immunity (2023) 56:2105–2120 e13. doi: 10.1016/j.immuni.2023.07.005 37527657

[108] Chung WS Clarke LE Wang GX Stafford BK Sher A Chakraborty C. Astrocytes mediate synapse elimination through MEGF10 and MERTK pathways. Nature (2013) 504:394–400. doi: 10.1038/nature12776 24270812 PMC3969024

[109] Lee JH Kim JY Noh S Lee H Lee SY Mun JY. Astrocytes phagocytose adult hippocampal synapses for circuit homeostasis. Nature (2021) 590:612–7. doi: 10.1038/s41586-020-03060-3 33361813

[110] Shi X Luo L Wang J Shen H Li Y Mamtilahun M. Stroke subtype-dependent synapse elimination by reactive gliosis in mice. Nat Commun (2021) 12:6943. doi: 10.1038/s41467-021-27248-x 34836962 PMC8626497

[111] Konishi H Okamoto T Hara Y Komine O Tamada H Maeda M. Astrocytic phagocytosis is a compensatory mechanism for microglial dysfunction. EMBO J (2020) 39:e104464. doi: 10.15252/embj.2020104464 32959911 PMC7667883

[112] Damisah EC Hill RA Rai A Chen F Rothlin CV Ghosh S. Astrocytes and microglia play orchestrated roles and respect phagocytic territories during neuronal corpse removal in vivo. Sci Adv (2020) 6:eaba3239. doi: 10.1126/sciadv.aba3239 32637606 PMC7319765

[113] Shinozaki Y Nomura M Iwatsuki K Moriyama Y Gachet C Koizumi S. Microglia trigger astrocyte-mediated neuroprotection via purinergic gliotransmission. Sci Rep (2014) 4:4329. doi: 10.1038/srep04329 24710318 PMC3948352

[114] Vainchtein ID Chin G Cho FS Kelley KW Miller JG Chien EC. Astrocyte-derived interleukin-33 promotes microglial synapse engulfment and neural circuit development. Science (2018) 359:1269–73. doi: 10.1126/science.aal3589 PMC6070131 29420261

[115] Griffiths I Klugmann M Anderson T Yool D Thomson C Schwab MH. Axonal swellings and degeneration in mice lacking the major proteolipid of myelin. Science (1998) 280:1610–3. doi: 10.1126/science.280.5369.1610 9616125

[116] Zhi JJ Wu SL Wu HQ Ran Q Gao X Chen JF. Insufficient oligodendrocyte turnover in optic nerve contributes to age-related axon loss and visual deficits. J Neurosci (2023) 43:1859–70. doi: 10.1523/JNEUROSCI.2130-22.2023 PMC10027114 36725322

[117] Son JL Soto I Oglesby E Lopez-Roca T Pease ME Quigley HA. Glaucomatous optic nerve injury involves early astrocyte reactivity and late oligodendrocyte loss. Glia (2010) 58:780–9. doi: 10.1002/glia.20962 20091782

[118] You Y Joseph C Wang C Gupta V Liu S Yiannikas C. Demyelination precedes axonal loss in the transneuronal spread of human neurodegenerative disease. Brain (2019) 142:426–42. doi: 10.1093/brain/awy338 30668642

[119] Nakazawa T Nakazawa C Matsubara A Noda K Hisatomi T She H. Tumor necrosis factor-alpha mediates oligodendrocyte death and delayed retinal ganglion cell loss in a mouse model of glaucoma. J Neurosci (2006) 26:12633–41. doi: 10.1523/JNEUROSCI.2801-06.2006 PMC6674838 17151265

[120] Baas D Legrand C Samarut J Flamant F. Persistence of oligodendrocyte precursor cells and altered myelination in optic nerve associated to retina degeneration in mice devoid of all thyroid hormone receptors. Proc Natl Acad Sci U.S.A. (2002) 99:2907–11. doi: 10.1073/pnas.052482299 PMC122446 11867729

[121] Gillow JT Shah P O’Neill EC. Primary open angle glaucoma and hypothyroidism: chance or true association? Eye (Lond) (1997) 11(Pt 1):113–4. doi: 10.1038/eye.1997.22 9246288

[122] McLenachan J Davies DM. Glaucoma and the thyroid. Br J Ophthalmol (1965) 49:441–4. doi: 10.1136/bjo.49.8.441 PMC506138 18170869

[123] Thvilum M Brandt F Brix TH Hegedus L. The interrelation between hypothyroidism and glaucoma: a critical review and meta-analyses. Acta Ophthalmol (2017) 95:759–67. doi: 10.1111/aos.13412 28211200

[124] Buchanan J Elabbady L Collman F Jorstad NL Bakken TE Ott C. Oligodendrocyte precursor cells ingest axons in the mouse neocortex. Proc Natl Acad Sci U.S.A. (2022) 119:e2202580119. doi: 10.1073/pnas.2202580119 36417438 PMC9889886

[125] Fernandez-Castaneda A Chappell MS Rosen DA Seki SM Beiter RM Johanson DM. The active contribution of OPCs to neuroinflammation is mediated by LRP1. Acta Neuropathol (2020) 139:365–82. doi: 10.1007/s00401-019-02073-1 PMC6994364 31552482

[126] Itoh N Itoh Y Tassoni A Ren E Kaito M Ohno A. Cell-specific and region-specific transcriptomics in the multiple sclerosis model: Focus on astrocytes. Proc Natl Acad Sci U.S.A. (2018) 115:E302–9. doi: 10.1073/pnas.1716032115 PMC5777065 29279367

[127] Ponath G Ramanan S Mubarak M Housley W Lee S Sahinkaya FR. Myelin phagocytosis by astrocytes after myelin damage promotes lesion pathology. Brain (2017) 140:399–413. doi: 10.1093/brain/aww298 28007993 PMC5841057

[128] Wan T Zhu W Zhao Y Zhang X Ye R Zuo M. Astrocytic phagocytosis contributes to demyelination after focal cortical ischemia in mice. Nat Commun (2022) 13:1134. doi: 10.1038/s41467-022-28777-9 35241660 PMC8894352

[129] Rosko L Smith VN Yamazaki R Huang JK. Oligodendrocyte bioenergetics in health and disease. Neuroscientist (2019) 25:334–43. doi: 10.1177/1073858418793077 PMC6745601 30122106

[130] Harun-Or-Rashid M Pappenhagen N Palmer PG Smith MA Gevorgyan V Wilson GN. Structural and functional rescue of chronic metabolically stressed optic nerves through respiration. J Neurosci (2018) 38:5122–39. doi: 10.1523/JNEUROSCI.3652-17.2018 PMC5977447 29760184

[131] Voskuhl RR Itoh N Tassoni A Matsukawa MA Ren E Tse V. Gene expression in oligodendrocytes during remyelination reveals cholesterol homeostasis as a therapeutic target in multiple sclerosis. Proc Natl Acad Sci U.S.A. (2019) 116:10130–9. doi: 10.1073/pnas.1821306116 PMC6525478 31040210

[132] Molina-Gonzalez I Holloway RK Jiwaji Z Dando O Kent SA Emelianova K. Astrocyte-oligodendrocyte interaction regulates central nervous system regeneration. Nat Commun (2023) 14:3372. doi: 10.1038/s41467-023-39046-8 37291151 PMC10250470

[133] Karim H Kim SH Lapato AS Yasui N Katzenellenbogen JA Tiwari-Woodruff SK. Increase in chemokine CXCL1 by ERbeta ligand treatment is a key mediator in promoting axon myelination. Proc Natl Acad Sci U.S.A. (2018) 115:6291–6. doi: 10.1073/pnas.1721732115 PMC6004485 29844175

[134] Gibson EM Purger D Mount CW Goldstein AK Lin GL Wood LS. Neuronal activity promotes oligodendrogenesis and adaptive myelination in the mammalian brain. Science (2014) 344:1252304. doi: 10.1126/science.1252304 24727982 PMC4096908

[135] Noori R Park D Griffiths JD Bells S Frankland PW Mabbott D. Activity-dependent myelination: A glial mechanism of oscillatory self-organization in large-scale brain networks. Proc Natl Acad Sci U.S.A. (2020) 117:13227–37. doi: 10.1073/pnas.1916646117 PMC7306810 32482855

[136] Lim JH Stafford BK Nguyen PL Lien BV Wang C Zukor K. Neural activity promotes long-distance, target-specific regeneration of adult retinal axons. Nat Neurosci (2016) 19:1073–84. doi: 10.1038/nn.4340 PMC5708130 27399843

[137] Wang D Tai PWL Gao G. Adeno-associated virus vector as a platform for gene therapy delivery. Nat Rev Drug Discovery (2019) 18:358–78. doi: 10.1038/s41573-019-0012-9 PMC6927556 30710128

[138] High KA Roncarolo MG. Gene therapy. N Engl J Med (2019) 381:455–64. doi: 10.1056/NEJMra1706910 31365802

[139] Russell S Bennett J Wellman JA Chung DC Yu ZF Tillman A. Efficacy and safety of voretigene neparvovec (AAV2-hRPE65v2) in patients with RPE65-mediated inherited retinal dystrophy: a randomised, controlled, open-label, phase 3 trial. Lancet (2017) 390:849–60. doi: 10.1016/S0140-6736(17)31868-8 PMC5726391 28712537

[140] Lee Y Messing A Su M Brenner M. GFAP promoter elements required for region-specific and astrocyte-specific expression. Glia (2008) 56:481–93. doi: 10.1002/glia.20622 18240313

[141] Klimczak RR Koerber JT Dalkara D Flannery JG Schaffer DV. A novel adeno-associated viral variant for efficient and selective intravitreal transduction of rat Muller cells. PloS One (2009) 4:e7467. doi: 10.1371/journal.pone.0007467 19826483 PMC2758586

[142] Koerber JT Klimczak R Jang JH Dalkara D Flannery JG Schaffer DV. Molecular evolution of adeno-associated virus for enhanced glial gene delivery. Mol Ther (2009) 17:2088–95. doi: 10.1038/mt.2009.184 PMC2788045 19672246

[143] Pellissier LP Hoek RM Vos RM Aartsen WM Klimczak RR Hoyng SA. Specific tools for targeting and expression in Muller glial cells. Mol Ther Methods Clin Dev (2014) 1:14009. doi: 10.1038/mtm.2014.9 26015954 PMC4362388

[144] Tanaka KF Matsui K Sasaki T Sano H Sugio S Fan K. Expanding the repertoire of optogenetically targeted cells with an enhanced gene expression system. Cell Rep (2012) 2:397–406. doi: 10.1016/j.celrep.2012.06.011 22854021

[145] Chen H McCarty DM Bruce AT Suzuki K Suzuki K. Gene transfer and expression in oligodendrocytes under the control of myelin basic protein transcriptional control region mediated by adeno-associated virus. Gene Ther (1998) 5:50–8. doi: 10.1038/sj.gt.3300547 9536264

[146] von Jonquieres G Frohlich D Klugmann CB Wen X Harasta AE Ramkumar R. Recombinant human myelin-associated glycoprotein promoter drives selective AAV-mediated transgene expression in oligodendrocytes. Front Mol Neurosci (2016) 9:13. doi: 10.3389/fnmol.2016.00013 26941604 PMC4763065

[147] Pollak M. The insulin and insulin-like growth factor receptor family in neoplasia: an update. Nat Rev Cancer (2012) 12:159–69. doi: 10.1038/nrc3215 22337149

[148] Agostinone J Alarcon-Martinez L Gamlin C Yu WQ Wong ROL Di Polo A. Insulin signalling promotes dendrite and synapse regeneration and restores circuit function after axonal injury. Brain (2018) 141:1963–80. doi: 10.1093/brain/awy142 PMC6022605 29931057

[149] Duan X Qiao M Bei F Kim IJ He Z Sanes JR. Subtype-specific regeneration of retinal ganglion cells following axotomy: effects of osteopontin and mTOR signaling. Neuron (2015) 85:1244–56. doi: 10.1016/j.neuron.2015.02.017 PMC4391013 25754821

[150] KermerPKlockerNLabesMBahrM. Insulin-like growth factor-I protects axotomized rat retinal ganglion cells from secondary death via PI3-K-dependent Akt phosphorylation and inhibition of caspase-3 In vivo. J Neurosci (2000) 20:2–8. doi: 10.1523/JNEUROSCI.20-02-00722.2000 10632601

[151] Zhang Y Williams PR Jacobi A Wang C Goel A Hirano AA. Elevating growth factor responsiveness and axon regeneration by modulating presynaptic inputs. Neuron (2019) 103:39–51 e5. doi: 10.1016/j.neuron.2019.04.033 31122676 PMC7350660

[152] Caldwell ALM Sancho L Deng J Bosworth A Miglietta A Diedrich JK. Aberrant astrocyte protein secretion contributes to altered neuronal development in multiple models of neurodevelopmental disorders. Nat Neurosci (2022) 25:1163–78. doi: 10.1038/s41593-022-01150-1 PMC10395413 36042312

[153] Liu X Yao DL Bondy CA Brenner M Hudson LD Zhou J. Astrocytes express insulin-like growth factor-I (IGF-I) and its binding protein, IGFBP-2, during demyelination induced by experimental autoimmune encephalomyelitis. Mol Cell Neurosci (1994) 5:418–30. doi: 10.1006/mcne.1994.1052 7529631

[154] Watanabe K Uemura K Asada M Maesako M Akiyama H Shimohama S. The participation of insulin-like growth factor-binding protein 3 released by astrocytes in the pathology of Alzheimer’s disease. Mol Brain (2015) 8:82. doi: 10.1186/s13041-015-0174-2 26637371 PMC4670528

[155] Lin R Zhou Y Yan T Wang R Li H Wu Z. Directed evolution of adeno-associated virus for efficient gene delivery to microglia. Nat Methods (2022) 19:976–85. doi: 10.1038/s41592-022-01547-7 35879607

[156] Okada Y Hosoi N Matsuzaki Y Fukai Y Hiraga A Nakai J. Development of microglia-targeting adeno-associated viral vectors as tools to study microglial behavior in vivo. Commun Biol (2022) 5:1224. doi: 10.1038/s42003-022-04200-3 36369525 PMC9652230

[157] Young A Neumann B Segel M Chen CZ Tourlomousis P Franklin RJM. Targeted evolution of adeno-associated virus capsids for systemic transgene delivery to microglia and tissue-resident macrophages. Proc Natl Acad Sci U.S.A. (2023) 120:e2302997120. doi: 10.1073/pnas.2302997120 37603759 PMC10469016

[158] Georgiou E Sidiropoulou K Richter J Papaneophytou C Sargiannidou I Kagiava A. Gene therapy targeting oligodendrocytes provides therapeutic benefit in a leukodystrophy model. Brain (2017) 140:599–616. doi: 10.1093/brain/aww351 28100454 PMC5837386

[159] Schiza N Sargiannidou I Kagiava A Karaiskos C Nearchou M Kleopa KA. Transgenic replacement of Cx32 in gap junction-deficient oligodendrocytes rescues the phenotype of a hypomyelinating leukodystrophy model. Hum Mol Genet (2015) 24:2049–64. doi: 10.1093/hmg/ddu725 25524707

[160] Elmore MR Najafi AR Koike MA Dagher NN Spangenberg EE Rice RA. Colony-stimulating factor 1 receptor signaling is necessary for microglia viability, unmasking a microglia progenitor cell in the adult brain. Neuron (2014) 82:380–97. doi: 10.1016/j.neuron.2014.02.040 PMC4161285 24742461

[161] Ebneter A Kokona D Jovanovic J Zinkernagel MS. Dramatic effect of oral CSF-1R kinase inhibitor on retinal microglia revealed by *in vivo* scanning laser ophthalmoscopy. Transl Vis Sci Technol (2017) 6:10. doi: 10.1167/tvst.6.2.10 PMC5407246 28458957

[162] Takeda A Shinozaki Y Kashiwagi K Ohno N Eto K Wake H. Microglia mediate non-cell-autonomous cell death of retinal ganglion cells. Glia (2018) 66:2366–84. doi: 10.1002/glia.23475 30375063

[163] Zhang Y Zhao L Wang X Ma W Lazere A Qian HH. Repopulating retinal microglia restore endogenous organization and function under CX3CL1-CX3CR1 regulation. Sci Adv (2018) 4:eaap8492. doi: 10.1126/sciadv.aap8492 29750189 PMC5943055

[164] Henry RJ Ritzel RM Barrett JP Doran SJ Jiao Y Leach JB. Microglial depletion with CSF1R inhibitor during chronic phase of experimental traumatic brain injury reduces neurodegeneration and neurological deficits. J Neurosci (2020) 40:2960–74. doi: 10.1523/JNEUROSCI.2402-19.2020 PMC7117897 32094203

[165] Rice RA Pham J Lee RJ Najafi AR West BL Green KN. Microglial repopulation resolves inflammation and promotes brain recovery after injury. Glia (2017) 65:931–44. doi: 10.1002/glia.23135 PMC5395311 28251674

[166] Rice RA Spangenberg EE Yamate-Morgan H Lee RJ Arora RP Hernandez MX. Elimination of microglia improves functional outcomes following extensive neuronal loss in the hippocampus. J Neurosci (2015) 35:9977–89. doi: 10.1523/JNEUROSCI.0336-15.2015 PMC4495246 26156998

[167] Wang Y Wernersbach I Strehle J Li S Appel D Klein M. Early posttraumatic CSF1R inhibition via PLX3397 leads to time- and sex-dependent effects on inflammation and neuronal maintenance after traumatic brain injury in mice. Brain Behav Immun (2022) 106:49–66. doi: 10.1016/j.bbi.2022.07.164 35933030

[168] George S Rey NL Tyson T Esquibel C Meyerdirk L Schulz E. Microglia affect alpha-synuclein cell-to-cell transfer in a mouse model of Parkinson’s disease. Mol Neurodegener (2019) 14:34. doi: 10.1186/s13024-019-0335-3 31419995 PMC6697982

[169] Olmos-Alonso A Schetters ST Sri S Askew K Mancuso R Vargas-Caballero M. Pharmacological targeting of CSF1R inhibits microglial proliferation and prevents the progression of Alzheimer’s-like pathology. Brain (2016) 139:891–907. doi: 10.1093/brain/awv379 26747862 PMC4766375

[170] Sosna J Philipp S Albay R 3rd Reyes-Ruiz JM Baglietto-Vargas D LaFerla FM. Early long-term administration of the CSF1R inhibitor PLX3397 ablates microglia and reduces accumulation of intraneuronal amyloid, neuritic plaque deposition and pre-fibrillar oligomers in 5XFAD mouse model of Alzheimer’s disease. Mol Neurodegener (2018) 13:11. doi: 10.1186/s13024-018-0244-x 29490706 PMC5831225

[171] Spangenberg EE Lee RJ Najafi AR Rice RA Elmore MR Blurton-Jones M. Eliminating microglia in Alzheimer’s mice prevents neuronal loss without modulating amyloid-beta pathology. Brain (2016) 139:1265–81. doi: 10.1093/brain/aww016 PMC5006229 26921617

[172] Okunuki Y Mukai R Nakao T Tabor SJ Butovsky O Dana R. Retinal microglia initiate neuroinflammation in ocular autoimmunity. Proc Natl Acad Sci U.S.A. (2019) 116:9989–98. doi: 10.1073/pnas.1820387116 PMC6525481 31023885

[173] Okunuki Y Mukai R Pearsall EA Klokman G Husain D Park DH. Microglia inhibit photoreceptor cell death and regulate immune cell infiltration in response to retinal detachment. Proc Natl Acad Sci U.S.A. (2018) 115:E6264–73. doi: 10.1073/pnas.1719601115 PMC6142210 29915052

[174] Yang X Zhao L Campos MM Abu-Asab M Ortolan D Hotaling N. CSF1R blockade induces macrophage ablation and results in mouse choroidal vascular atrophy and RPE disorganization. Elife (2020) 9:e55564. doi: 10.7554/eLife.55564 PMC7156269 32234210

[175] Hilla AM Diekmann H Fischer D. Microglia are irrelevant for neuronal degeneration and axon regeneration after acute injury. J Neurosci (2017) 37:6113–24. doi: 10.1523/JNEUROSCI.0584-17.2017 PMC6596505 28539419

[176] Elmore MRP Hohsfield LA Kramar EA Soreq L Lee RJ Pham ST. Replacement of microglia in the aged brain reverses cognitive, synaptic, and neuronal deficits in mice. Aging Cell (2018) 17:e12832. doi: 10.1111/acel.12832 30276955 PMC6260908

[177] Diemler CA Cossete T Heuer S Goodrich MM MacLean M Marola O. Microglia depletion increases susceptibility for glaucomatous neurodegeneration in ocular hypertensive mice. ARVO Annu Meeting Abstract (2023) 64:1595.

[178] Tan Z Guo Y Shrestha M Sun D Gregory-Ksander M Jakobs TC. Microglia depletion exacerbates retinal ganglion cell loss in a mouse model of glaucoma. Exp Eye Res (2022) 225:109273. doi: 10.1016/j.exer.2022.109273 36206859 PMC10970711

[179] Parajuli B Saito H Shinozaki Y Shigetomi E Miwa H Yoneda S. Transnasal transplantation of human induced pluripotent stem cell-derived microglia to the brain of immunocompetent mice. Glia (2021) 69:2332–48. doi: 10.1002/glia.23985 34309082

[180] Parajuli B Shinozaki Y Shigetomi E Koizumi S. Transplantation of human induced pluripotent stem cell-derived microglia in immunocompetent mice brain via non-invasive transnasal route. J Vis Exp (2022) 183. doi: 10.3791/63574-v 35723460

[181] Tvrdik P Kalani MYS. *In vivo* imaging of microglial calcium signaling in brain inflammation and injury. Int J Mol Sci (2017) 18:2366. doi: 10.3390/ijms18112366 29117112 PMC5713335

[182] Ma W Xhao L Xu B Fariss RN Redmond TM Zou J. Human iPSC-derived Microglia Cells Integrated into Mouse Retina and Recapitulated Features of Endogenous Microglia Cells. bioRxiv (2023). doi: 10.7554/eLife.90695.1

[183] Livne-Bar I Wei J Liu HH Alqawlaq S Won GJ Tuccitto A. Astrocyte-derived lipoxins A4 and B4 promote neuroprotection from acute and chronic injury. J Clin Invest (2017) 127:4403–14. doi: 10.1172/JCI77398 PMC5707141 29106385

[184] Bull ND Irvine KA Franklin RJ Martin KR. Transplanted oligodendrocyte precursor cells reduce neurodegeneration in a model of glaucoma. Invest Ophthalmol Vis Sci (2009) 50:4244–53. doi: 10.1167/iovs.08-3239 19357352

[185] Ader M Meng J Schachner M Bartsch U. Formation of myelin after transplantation of neural precursor cells into the retina of young postnatal mice. Glia (2000) 30:301–10. doi: 10.1002/(SICI)1098-1136(200005)30:3<301::AID-GLIA9>3.0.CO;2-S 10756079

[186] Laeng P Molthagen M Yu EG Bartsch U. Transplantation of oligodendrocyte progenitor cells into the rat retina: extensive myelination of retinal ganglion cell axons. Glia (1996) 18:200–10. doi: 10.1002/(SICI)1098-1136(199611)18:3<200::AID-GLIA4>3.0.CO;2-2 8915652

[187] Yang X Zou H Jung G Richard G Linke SJ Ader M. Nonneuronal control of the differential distribution of myelin along retinal ganglion cell axons in the mouse. Invest Ophthalmol Vis Sci (2013) 54:7819–27. doi: 10.1167/iovs.13-12596 24222305

[188] Fujikado T Morimoto T Matsushita K Shimojo H Okawa Y Tano Y. Effect of transcorneal electrical stimulation in patients with nonarteritic ischemic optic neuropathy or traumatic optic neuropathy. Jpn J Ophthalmol (2006) 50:266–73. doi: 10.1007/s10384-005-0304-y 16767383

[189] Morimoto T Miyoshi T Sawai H Fujikado T. Optimal parameters of transcorneal electrical stimulation (TES) to be neuroprotective of axotomized RGCs in adult rats. Exp Eye Res (2010) 90:285–91. doi: 10.1016/j.exer.2009.11.002 19909741

[190] Yin H Yin H Zhang W Miao Q Qin Z Guo S. Transcorneal electrical stimulation promotes survival of retinal ganglion cells after optic nerve transection in rats accompanied by reduced microglial activation and TNF-alpha expression. Brain Res (2016) 1650:10–20. doi: 10.1016/j.brainres.2016.08.034 27569587

[191] Inomata K Shinoda K Ohde H Tsunoda K Hanazono G Kimura I. Transcorneal electrical stimulation of retina to treat longstanding retinal artery occlusion. Graefes Arch Clin Exp Ophthalmol (2007) 245:1773–80. doi: 10.1007/s00417-007-0610-9 17593383

[192] Wang X Mo X Li D Wang Y Fang Y Rong X. Neuroprotective effect of transcorneal electrical stimulation on ischemic damage in the rat retina. Exp Eye Res (2011) 93:753–60. doi: 10.1016/j.exer.2011.09.022 22008240

[193] Ni YQ Gan DK Xu HD Xu GZ Da CD. Neuroprotective effect of transcorneal electrical stimulation on light-induced photoreceptor degeneration. Exp Neurol (2009) 219:439–52. doi: 10.1016/j.expneurol.2009.06.016 19576889

[194] Yu H Enayati S Chang K Cho K Lee SW Talib M. Noninvasive electrical stimulation improves photoreceptor survival and retinal function in mice with inherited photoreceptor degeneration. Invest Ophthalmol Vis Sci (2020) 61:5. doi: 10.1167/iovs.61.4.5 PMC7401948 32271885

[195] Jassim AH Cavanaugh M Shah JS Willits R Inman DM. Transcorneal electrical stimulation reduces neurodegenerative process in a mouse model of glaucoma. Ann BioMed Eng (2021) 49:858–70. doi: 10.1007/s10439-020-02608-8 PMC7854493 32974756

[196] Ota Y Ozeki N Yuki K Shiba D Kimura I Tsunoda K. The efficacy of transcorneal electrical stimulation for the treatment of primary open-angle glaucoma: A pilot study. Keio J Med (2018) 67:45–53. doi: 10.2302/kjm.2017-0015-OA 29415904

[197] Fu L Fung FK Lo AC Chan YK So KF Wong IY. Transcorneal electrical stimulation inhibits retinal microglial activation and enhances retinal ganglion cell survival after acute ocular hypertensive injury. Transl Vis Sci Technol (2018) 7:7. doi: 10.1167/tvst.7.3.7 PMC5976234 29862139

[198] Zhou WT Ni YQ Jin ZB Zhang M Wu JH Zhu Y. Electrical stimulation ameliorates light-induced photoreceptor degeneration in *vitro* via suppressing the proinflammatory effect of microglia and enhancing the neurotrophic potential of Muller cells. Exp Neurol (2012) 238:192–208. doi: 10.1016/j.expneurol.2012.08.029 22974557

[199] Stett A Schatz A Gekeler F Franklin J. Transcorneal electrical stimulation dose-dependently slows the visual field loss in retinitis pigmentosa. Transl Vis Sci Technol (2023) 12:29. doi: 10.1167/tvst.12.2.29 PMC9946045 36809335

[200] Miura G Sugawara T Kawasaki Y Tatsumi T Nizawa T Baba T. Clinical trial to evaluate safety and efficacy of transdermal electrical stimulation on visual functions of patients with retinitis pigmentosa. Sci Rep (2019) 9:11668. doi: 10.1038/s41598-019-48158-5 31406205 PMC6690905

[201] Schatz A Rock T Naycheva L Willmann G Wilhelm B Peters T. Transcorneal electrical stimulation for patients with retinitis pigmentosa: a prospective, randomized, sham-controlled exploratory study. Invest Ophthalmol Vis Sci (2011) 52:4485–96. doi: 10.1167/iovs.10-6932 21467183

[202] van Niel G D’Angelo G Raposo G. Shedding light on the cell biology of extracellular vesicles. Nat Rev Mol Cell Biol (2018) 19:213–28. doi: 10.1038/nrm.2017.125 29339798

[203] Elliott RO He M. Unlocking the power of exosomes for crossing biological barriers in drug delivery. Pharmaceutics (2021) 13:122. doi: 10.3390/pharmaceutics13010122 33477972 PMC7835896

[204] Aires ID Ribeiro-Rodrigues T Boia R Catarino S Girao H Ambrosio AF. Exosomes derived from microglia exposed to elevated pressure amplify the neuroinflammatory response in retinal cells. Glia (2020) 68:2705–24. doi: 10.1002/glia.23880 32645245

[205] Carapia AK Martinez-Colin EJ Segura-Villalobos D Victoria-Chavez RY Lezama I Martinez-Martinez E. Muller glia to muller glia extracellular vesicle-dependent signaling induces multipotency genes nestin and lin28 expression in response to N-methyl-D-aspartate (NMDA) exposure. ASN Neuro (2023) 15:17590914231183272. doi: 10.1177/17590914231183272 37345290 PMC10291543

[206] Demais V Pohl A Wunderlich KA Pfaller AM Kaplan L Barthelemy A. Release of VAMP5-positive extracellular vesicles by retinal Muller glia in vivo. J Extracell Vesicles (2022) 11:e12254. doi: 10.1002/jev2.12254 36043482 PMC9428896

[207] Salikhova DI Timofeeva AV Golovicheva VV Fatkhudinov TK Shevtsova YA Soboleva AG. Extracellular vesicles of human glial cells exert neuroprotective effects via brain miRNA modulation in a rat model of traumatic brain injury. Sci Rep (2023) 13:20388. doi: 10.1038/s41598-023-47627-2 37989873 PMC10663567

[208] Gao Y Li H Qin C Yang B Ke Y. Embryonic stem cells-derived exosomes enhance retrodifferentiation of retinal Muller cells by delivering BDNF protein to activate Wnt pathway. Immunobiology (2022) 227:152211. doi: 10.1016/j.imbio.2022.152211 35390666

